# Subnetwork-Specific Homeostatic Plasticity in Mouse Visual Cortex In Vivo

**DOI:** 10.1016/j.neuron.2015.05.010

**Published:** 2015-06-03

**Authors:** Samuel J. Barnes, Rosanna P. Sammons, R. Irene Jacobsen, Jennifer Mackie, Georg B. Keller, Tara Keck

**Affiliations:** 1 MRC Centre for Developmental Neurobiology, King’s College London, New Hunt’s House 4^th^ Floor, London SE1 1UL, UK; 2 Department of Neuroscience, Physiology, and Pharmacology, University College London, 21 University Street, London WC1E 6DE, UK; 3 Friedrich Miescher Institute for Biomedical Research, Maulbeerstrasse 66, Basel 4058, Switzerland

## Abstract

Homeostatic regulation has been shown to restore cortical activity in vivo following sensory deprivation, but it is unclear whether this recovery is uniform across all cells or specific to a subset of the network. To address this issue, we used chronic calcium imaging in behaving adult mice to examine the activity of individual excitatory and inhibitory neurons in the same region of the layer 2/3 monocular visual cortex following enucleation. We found that only a fraction of excitatory neurons homeostatically recover activity after deprivation and inhibitory neurons show no recovery. Prior to deprivation, excitatory cells that did recover were more likely to have significantly correlated activity with other recovering excitatory neurons, thus forming a subnetwork of recovering neurons. These network level changes are accompanied by a reduction in synaptic inhibition onto all excitatory neurons, suggesting that both synaptic mechanisms and subnetwork activity are important for homeostatic recovery of activity after deprivation.

## Highlights

• In adult cortex, a subset of excitatory neurons recover activity after enucleation
• Inhibitory neurons do not homeostatically recover activity over 72 hr
• Excitatory neurons recover in subnetwork specific manner
• Recovery of activity is facilitated by reduced synaptic inhibition

## Teaser

Barnes et al. examine homeostatic recovery of activity of individual excitatory and inhibitory neurons in the adult cortex following enucleation. A fraction of excitatory neurons recover activity, in a subnetwork specific manner, but inhibitory cells do not recover activity over 72 hr.

## Introduction

Homeostatic plasticity is thought to be essential for maintaining the firing rate of neurons and preventing aberrantly low or high network activity (Turrigiano and Nelson, 2004). Homeostatic restoration of cortical activity following sensory deprivation has been demonstrated in vivo in the visual cortex in excitatory cells in both the developing (Hengen et al., 2013) and adult (Keck et al., 2013) rodent. Multiple homeostatic mechanisms that may underlie activity restoration following sensory deprivation have been described, including synaptic scaling, alterations to the balance between excitation and inhibition (E/I balance), and changes to neuronal excitability (Turrigiano, 2011). Ex vivo experiments performed during development have shown that different sensory deprivation paradigms invoke different homeostatic mechanisms in neuronal circuits of cortical layer 2/3 (L2/3) (Maffei and Turrigiano, 2008). Furthermore, these mechanisms are expressed in a layer specific manner at different stages of development (Desai et al., 2002; Goel and Lee, 2007; Lambo and Turrigiano, 2013).

In the adult cortex, excitatory plasticity is often preceded by a reduction in synaptic inhibition onto excitatory cells (Chen et al., 2011; Keck et al., 2011; Kuhlman et al., 2013; van Versendaal et al., 2012), which modifies the normal E/I balance (Xue et al., 2014). The relative time course of these changes, as well as the fact that they occur following a number of different deprivation paradigms, including monocular deprivation, focal retinal lesions, and binocular retinal lesions, suggests that reduced synaptic inhibition may generally be permissive for excitatory reorganization (Chen et al., 2011). Consistent with this idea, the artificial reduction of inhibition in vivo increases cortical plasticity (Chen et al., 2011; Fu et al., 2015; Hensch et al., 1998). Reduced activity in the inhibitory cells themselves also plays a role in plasticity in the developing cortex (Kuhlman et al., 2013), and this reduced inhibitory cell activity recovers following sensory deprivation via monocular deprivation (Hengen et al., 2013). It remains unclear whether recovery of inhibitory cell activity occurs in adult animals, which, given that inhibitory cells are extensively connected to local excitatory cells (Fino and Yuste, 2011; Hofer et al., 2011), will have important implications for local network activity.

Evidence suggests that cortical networks organize into smaller subnetworks of connected neurons within the wider network of excitatory and inhibitory cells (Harris and Mrsic-Flogel, 2013). These subnetworks express a variety of different connectivity patterns ranging from very local connectivity (Kozloski et al., 2001; Miller et al., 2014; Song et al., 2005) to intra-laminar connectivity (Kätzel et al., 2011; Yoshimura et al., 2005). Excitatory neurons form subnetworks of connected neurons that share a common organizing principle, such as preferences for sensory stimuli (Ko et al., 2011), intra-laminar inputs (Yoshimura et al., 2005), projection targets (Brown and Hestrin, 2009), or progenitors (Yu et al., 2009). Recent evidence suggests that inhibitory neurons form connections in a more widespread and less specific manner than excitatory cells (Fino and Yuste, 2011; Hofer et al., 2011). Thus, the role of inhibitory neurons in subnetworks likely differs from that of excitatory cells (Fino and Yuste, 2011; Hofer et al., 2011; Yoshimura and Callaway, 2005). Furthermore, the role of subnetworks during cortical plasticity following sensory deprivation is unclear.

To investigate the role of subnetworks of cells in homeostatic plasticity, we first measured the responses of individual excitatory and inhibitory neurons in the same cortical region of the monocular visual cortex in adult mice following monocular enucleation. We found that individual excitatory neurons in L2/3 could be divided into two groups: (1) those that become completely inactive after deprivation and (2) those that homeostatically recover activity, i.e., those in which a period of reduced activity is followed by a gradual recovery of activity over a period of 48–72 hr. In contrast, inhibitory neurons either become completely inactive or exhibit decreased activity, but do not show recovery of activity. When we examined known homeostatic mechanisms, we found that following deprivation, synaptic inhibition was reduced onto both putative recovering and inactive L2/3 excitatory neurons.

Finally, when we investigated the role of subnetworks in homeostatic recovery, we found that, prior to deprivation, recovering neurons were more likely to have had correlated activity with other recovering excitatory neurons and inhibitory neurons that remain active. Similarly, prior to enucleation, cells that would become inactive after deprivation were more often correlated with other cells that would become inactive, suggesting that there are subnetworks of neurons that will either recover or become inactive. These results indicate that in L2/3 of the adult visual cortex, homeostatic recovery of activity involves both network activity and a reduction in synaptic inhibition.

## Results

### Classification of Excitatory and Inhibitory Neurons in Behaving Animals

**Figure 1:**
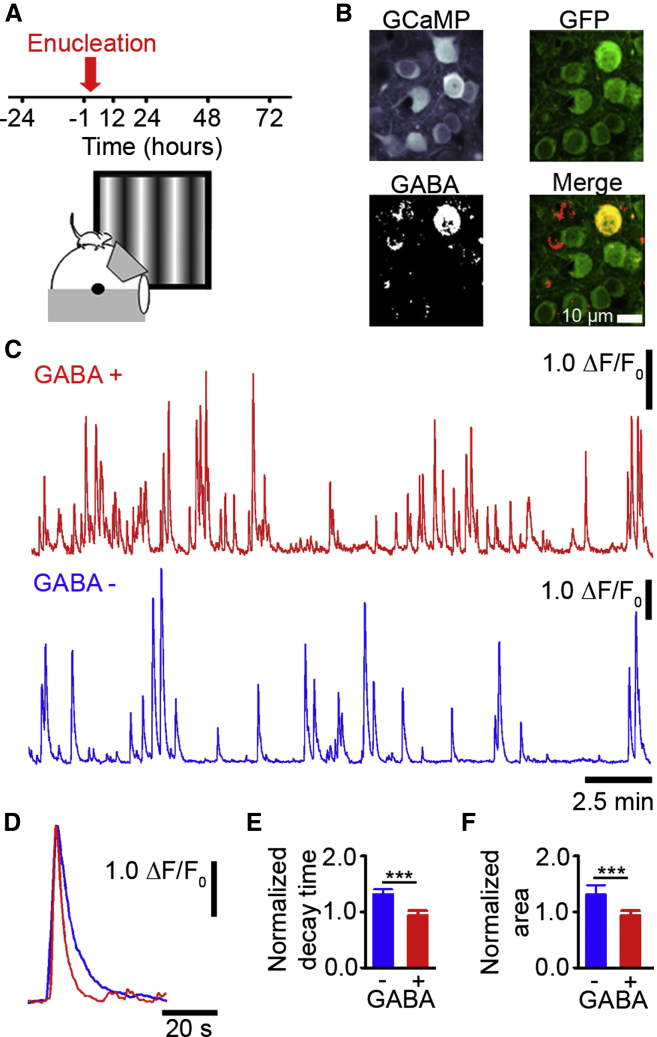
Classification of GABA-Positive and GABA-Negative Neurons (A) The top shows experimental time course, and the bottom shows a schematic of the experimental setup. (B) Example GCaMP5 expressing neurons in vivo (upper left), same neurons after sectioning and reconstruction, with immunolabeled GFP (upper right) or GABA (bottom left) and merge of GABA and GFP images (bottom right). (C and D) Example calcium transients from a GABA-positive (putative inhibitory, red) and a GABA-negative neuron (putative excitatory, blue). (E) Mean normalized calcium transient decay time (p < 0.001 and t test). (F) Mean normalized calcium transient total area (p < 0.001 and t test). (E and F) Calcium transients are normalized to the mean of all cells in the animal (^∗∗∗^p < 0.001). The error bars represent SEM. See also Figure S1.

We used the genetically encoded calcium indicator GCaMP5 (Akerboom et al., 2012) to chronically measure the calcium signals of individual neurons in L2/3 of the adult monocular visual cortex in behaving mice on a spherical treadmill (Figure 1A; Keck et al., 2013). Neurons in the same region of the visual cortex were repeatedly imaged both in the 24 hr before and the 72 hr after monocular enucleation (Figure 1A), with the intent to examine homeostatic changes in individual cells. Because we hypothesized that excitatory and inhibitory cells may respond differently to sensory deprivation, we first set out to identify which of the neurons we imaged were excitatory and which were GABAergic, so that we could determine their relative roles in homeostatic plasticity. Thus, upon completion of the imaging time course, each brain was sectioned parallel to the imaging plane and immunolabelled using antibodies for both GFP, to enhance the GCaMP5 signal, and the inhibitory neurotransmitter GABA, to identify inhibitory neurons (Figure 1B). The cortical regions imaged in vivo were reconstructed in histological sections and the previously imaged cells were identified as putative excitatory neurons or putative inhibitory neurons based on the immunohistochemistry (Figure 1B; see Kerlin et al., 2010; see Supplemental Information). This allowed us to examine the calcium traces of identified cell types (Figure 1C).

We measured the kinetics of calcium transients prior to deprivation and found cell type differences (Figures 1C and 1D). The kinetics of isolated calcium transients from inhibitory neurons were faster than those from excitatory neurons. They had shorter normalized decay times (Figure 1E) and a smaller total area (Figure 1F). Given these differences, we developed a classifier to separate excitatory and inhibitory cell types based on their calcium transients. We first trained this classifier on half of our data in which the excitatory and inhibitory cells were identified to determine the thresholds for separating cell types. We then cross-validated the classifier on the second half of the data, which was novel to the classifier and found it could separate cell types based on calcium transients alone with 91% accuracy (Figure S1, see Experimental Procedures). This allowed us to study the activity profiles of the same excitatory and inhibitory neurons, as well as their interactions, in vivo before and after monocular enucleation.

### Recovery of Activity Occurs in a Subset of Excitatory Neurons

Having classified neurons into excitatory and inhibitory cell types, we chronically measured the effect of monocular enucleation using calcium signals as a proxy for neuronal activity in putative excitatory cells. We first measured the average activity of all cells and found that, similar to binocular retinal lesions (Keck et al., 2013) and monocular deprivation (Hengen et al., 2013), activity decreases following enucleation and recovers over 48–72 hr (Figure S2A, see Discussion). To determine if this recovery of activity occurs in all of the neurons, we analyzed the activity profiles of individual neurons.

First, we examined whether all visually driven neurons that were active before enucleation undergo homeostatic recovery of activity. We measured excitatory cells that were visually responsive (i.e., were significantly more responsive during visual stimulation than in darkness, in the absence of locomotion) prior to enucleation. The activity of these cells accounted for 87% of all the activity measured in our paradigm prior to (sham) enucleation. Consistent with previous work (Peters et al., 2014), we found that whether or not a cell was active on a given day was variable: the measured day-to-day variability of the population in control animals (i.e., the fraction of cells that become inactive/active between two 15 min recording sessions spaced by 24 hr) was 25% ± 1% for cells becoming inactive and 19% ± 4% for cells becoming active.

**Figure 2:**
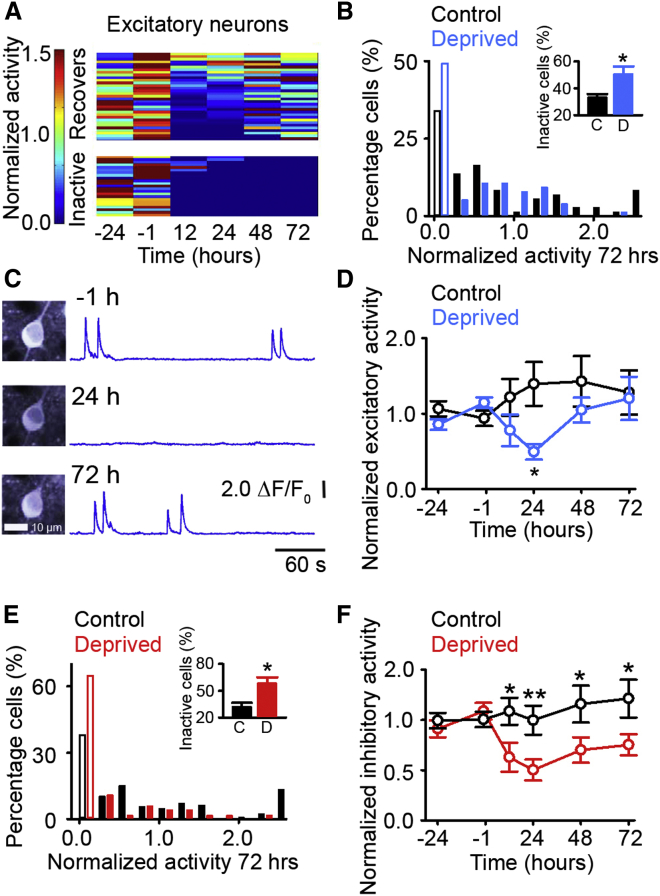
A Subset of Cortical Excitatory Neurons Recover Activity (A) Activity of representative examples of individual excitatory neurons (each horizontal line represents the activity of a single neuron) in enucleated animals, normalized in individual cells by the average baseline activity (−24 and −1 hr). (B) Distribution of activity of visually responsive excitatory neurons 72 hr after enucleation (blue) or control (black) normalized in individual cells to the average baseline activity. The cells either become inactive (open bars) or exhibit some degree of recovery (filled bars). The inset shows the average fraction of inactive cells per animal (control [black, n = 4 animals, and SD = 0.045] versus enucleation [blue, n = 4 animals, and SD = 0.114] p = 0.031 and t test). (C) Representative example of a recovering excitatory neuron’s activity. The left shows the maximum intensity projections for the same cell. The right shows ΔF/F_0_ signals for the neuron prior to, 24, and 72 hr after enucleation. (D) Mean activity of recovering excitatory neurons (not including inactive cells) normalized in individual cells to average baseline activity (control versus enucleation: 12 hr and p = 0.072; 24 hr and p = 0.012; 48 hr and p = 0.274; and 72 hr, p = 0.853, and t test). (E) Distribution of activity of visually responsive inhibitory neurons 72 hr after enucleation (red) or control (black) normalized in individual cells to average baseline activity. The cells either become inactive (open bars) or have some level of activity (filled bars). The inset shows the average fraction of inactive cells per animal (control [black, n = 4 animals, and SD = 0.081] versus enucleation [red, n = 4 animals, and SD = 0.137] p = 0.017 and t test). (F) Mean activity of active inhibitory neurons (not including inactive cells) normalized in individual cells to average baseline activity (control versus enucleation: 12 hr and p = 0.014; 24 hr and p < 0.001; 48 hr and p = 0.030; and 72 hr, p = 0.028, and t test) (^∗^p < 0.05 and ^∗∗^p < 0.01). The error bars represent SEM. See also Figure S2.

Following deprivation, we found that we could separate excitatory cells that were active during baseline into two groups: either (1) becoming inactive after deprivation—i.e., having no activity 72 hr after enucleation—which we will refer to as “inactive” or (2) recovering their activity—i.e., are active 72 hr after deprivation—which we will refer to as “recovering” (Figure 2A). After enucleation, 48% of cells became inactive (Figure 2B) and thus do not contribute to the homeostatic recovery observed in the average activity. There was a significantly higher fraction of these inactive cells in enucleated animals relative to cells that became inactive over the same time course in control animals (Figure 2B, inset; 32% of cells). The other 52% of excitatory cells recovered their activity after enucleation and, on average, exhibited an activity profile (Figures 2C and 2D) where activity decreased rapidly (12 to 24 hr) after enucleation and then gradually recovered (Figure 2D).

We next examined whether the homeostatic restoration observed is partially attributable to the recruitment of cells that were inactive prior to deprivation, as has previously been described following deprivation of L2/3 neurons in somatosensory cortex (Margolis et al., 2012). We found that, in our paradigm, only a small percentage of excitatory neurons that were previously inactive during both baseline time points (−24 and −1 hr) became active after 72 hr in control animals (4% ± 1%) or following enucleation (3% ± 1%; control versus enucleation, p = 0.452, t test). Furthermore, the overall activity levels of these neurons were very low, accounting for less than 5% of the total activity 72 hr after (sham) enucleation. Thus, these data indicate that only a subset of cells undergo homeostatic recovery of activity following visual deprivation and that the recovery is not driven by the recruitment of cells that were previously inactive.

We then examined if homeostatic recovery could be explained by differences in the input properties of recovering or inactive cells. We specifically examined visual activity and locomotion-related drive, as cells in the visual cortex are modulated by locomotion (Andermann et al., 2011; Ayaz et al., 2013; Keller et al., 2012; Niell and Stryker, 2010; Saleem et al., 2013). In activity measurements prior to enucleation, we found no difference between recovering and inactive cells in overall baseline activity (Figure S4A), orientation selectivity (Figure S2B), orientation tuning preference (percentage of cells [calculated per animal] tuned to 0 degrees: recovering 25% ± 8%, inactive 29% ± 6%, and p = 0.913; 45 degrees: recovering 31% ± 11%, inactive 19% ± 8%, and p = 0.389; 90 degrees: recovering 13% ± 11%, inactive 12% ± 8%, and p = 0.388; 135 degrees: recovering 31% ± 19%, inactive 40% ± 10%, and p = 0.848, t test), or responses to locomotion in the darkness (Figure S2C). Furthermore, responses following enucleation were not visually evoked, as their occurrence at the onset (within 500 ms) of visual stimuli was similar to chance levels (Figure S2D) and responses were equally likely to occur in light and dark conditions after enucleation (Figure S2E). These results suggest that the spared ipsilateral eye was not directly driving the recovering cells. While we found no evidence for common inputs to recovering or inactive cells, when we measured cortical activity while the mice were stationary in the dark, as a proxy for spontaneous activity, we saw that spontaneous activity was maintained and subsequently increased following enucleation in the recovering neurons, but not in the cells that became inactive (Figure S2F). Finally, we found no effects on the cellular properties of cells expressing GCaMP5 or a difference in GCaMP5 expression in recovering or inactive cells (Figures S2G–S2O).

Having characterized the activity of putative excitatory neurons, we then examined the activity in putative inhibitory neurons, which has previously only been measured following sensory deprivation during development (Hengen et al., 2013). We found a reduction in inhibitory cell activity following enucleation, with approximately 65% of inhibitory neurons becoming inactive (having no activity 72 hr after deprivation), in comparison to 38% of cells that no longer responded over the same time course in control mice (Figure 2E). Those inhibitory neurons that remained active 72 hr after deprivation exhibited a reduction in normalized activity 12 hr after enucleation (Figure 2F) and unlike the excitatory neurons, the activity of these inhibitory neurons did not show a significant recovery over time (Figure 2F). We observed no difference in cellular properties (Figures S2G, S2I, and S2P–S2U) or plasticity (Figures S2V–S2Y) in GCaMP5-positive and -negative inhibitory neurons, suggesting this lack of recovery is unrelated to the expression of our calcium indicator. To ensure that there was not an increase in inhibitory activity in the cortex via the post-enucleation addition of previously silent inhibitory neurons, we measured the number of newly activated inhibitory neurons 72 hr after enucleation that had not been active in either baseline time point (−24 and −1 hr) and found that it was low in both control mice (2% ± 1%) and following enucleation (3% ± 2%; control versus enucleation, p = 0.537, t test). Together, these results suggest that a subset of inhibitory cells remain active, but that their activity levels are decreased, implicating that there are overall lower levels of cortical inhibition. Thus, we show for the first time that inhibitory neurons in adult animals do not homeostatically recover their activity over the time course of 72 hr.

### Synaptic Inhibition Is Decreased onto L2/3 Excitatory Cells after Deprivation

**Figure 3:**
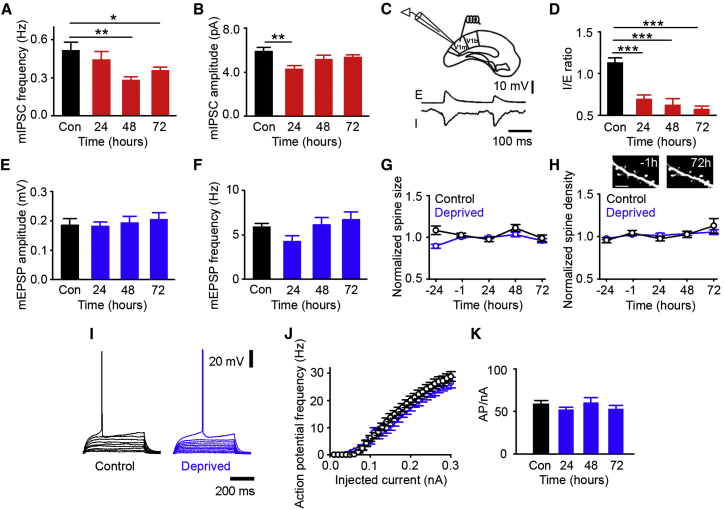
Synaptic Inhibition Is Reduced after Enucleation (A and B) Average mIPSC frequency (A) or amplitude (B) (control [n = 9] versus enucleation: A, 24 hr, p = 0.300, and n = 9; 48 hr, p = 0.004, and n = 7; 72 hr, p = 0.033, and n = 9; B, 24 hr, p = 0.002, and n = 9; 48 hr, p = 0.180, and n = 7; and 72 hr, p = 0.168, n = 9, and ANOVA with post hoc test). (C) The top shows a schematic of acute slice preparation with recording and stimulating electrodes in L2/3 (monocular visual cortex [V1m] and binocular visual cortex [V1b]). The bottom shows example excitatory (E) and inhibitory (I) traces for the same control cell, acquired by holding the neuron at −70 and +10 mV, respectively. (D) Ratio of inhibitory to excitatory evoked transient amplitude (control [n = 15] versus enucleation, 24 hr, p < 0.001, and n = 10; 48 hr, p < 0.001, and n = 8; and 72 hr, p < 0.001, n = 14, and ANOVA with post hoc test). (E and F) Average mEPSP amplitude (E) or frequency (F) (control [n = 11] versus enucleation, 24 hr, and n = 10; 48 hr and n = 11; 72 hr and n = 12; E, p = 0.832; and F, p = 0.107 and ANOVA). (G) Stable spine size following enucleation (blue) or in control (black) normalized in individual spines by baseline average (control [n = 82 spines] versus enucleation [n = 239 spines]: 24 hr and p = 0.400; 48 hr and p = 0.142; and 72 hr, p = 0.553, and t test). (H) Spine density normalized by baseline average in individual dendritic branches. (Control [black, n = 11 branches] versus enucleation [blue, n = 20 branches], 24 hr and p = 0.347; 48 hr and p = 0.827; and 72 hr, p = 0.328, and t test). The inset shows an example of the same dendritic branch 1 hr before and 72 hr after enucleation. The scale bar represents 5 μm. (I) Example voltage response to a series of somatic current injections (0 to 0.09 nanoAmpere [nA]) 72 hr after (sham) enucleation. (J) Input-output function: injected current and resulting average action potential (AP) frequency 72 hr after enucleation (blue) or in control (black). (K) Average number of APs per nA of injected current calculated as the slope of the input-output function measured for each cell after enucleation (blue) or in control (black) (control [n = 21] versus enucleation, 24 hr, and n = 12; 48 hr and n = 11; 72 hr and n = 16; and p = 0.492 and ANOVA). All measurements are made in L2/3 excitatory cells (^∗^p < 0.05, ^∗∗^p < 0.01, and ^∗∗∗^p < 0.001). The error bars represent SEM.

Having described homeostatic recovery in a subset of excitatory, but not inhibitory, cells, we next examined if there are changes in identified homeostatic mechanisms in excitatory cells following deprivation and the time course of these changes. We first checked for changes in synaptic inhibition onto the L2/3 excitatory cells by performing whole-cell patch clamp recordings in acute slices prepared from mice that had previously undergone monocular enucleation. We measured the frequency and amplitude of miniature inhibitory post-synaptic currents (mIPSCs), which approximate the number and strength, respectively, of the inhibitory synapses onto these cells. Similar to previous studies in layer 5 (L5) cells (Keck et al., 2011, 2013), we found that mIPSC frequency (Figure 3A), but not amplitude (Figure 3B), was significantly and consistently decreased, suggesting that there are fewer inhibitory synapses onto these L2/3 cells. We next examined changes in evoked inhibitory potentials by measuring the balance between evoked (with the stimulating electrode in L2/3) excitatory and inhibitory potentials on L2/3 cells 24, 48, or 72 hr after deprivation (Figure 3C). We found the E/I balance to be shifted toward excitation in individual L2/3 excitatory neurons 24 hr after enucleation (Figure 3D), prior to the observed recovery of excitatory cortical activity 48 hr after deprivation. Together, these data suggest that synaptic inhibition is decreased, which may play a role in facilitating the homeostatic recovery in activity levels.

We next examined if synaptic scaling occurs after monocular enucleation. Over our 72 hr time course, we found no change in either miniature excitatory post-synaptic potential (mEPSP) amplitude (Figure 3E) or frequency (Figure 3F) in acute slices prepared from mice that had previously undergone enucleation. Then, in a separate subset of mice that express GFP under the *thy-1* promoter (GFP-M line, Feng et al., 2000), we used chronic two-photon structural imaging to repeatedly measure dendritic spines before and after deprivation. We calculated spine size and density as a proxy for synaptic scaling in vivo (see Keck et al., 2013) and found no change in either spine size (Figure 3G) or density (Figure 3H) after deprivation in L2/3 cells. In comparison to previous work that observed synaptic scaling in L5 neurons following sensory deprivation via retinal lesions in the adult visual cortex (Keck et al., 2013), these data suggest that synaptic scaling is not likely to be driving homeostatic recovery in L2/3. Taken together, these results indicate that homeostatic mechanisms may be layer specific in the adult cortex. We finally checked the intrinsic excitability of L2/3 pyramidal cells measured as the relationship between the input current and the resulting number of action potentials generated in excitatory neurons in acute slices prepared from enucleated mice 72 hr after deprivation. We found no change in the average action potential frequency after deprivation (Figures 3I–3K) or the action potential threshold (current necessary to induce a single action potential, Figure 3J). Similarly, the passive membrane properties, which may influence excitability, were not modified by enucleation (Table 1). Taken together, these results suggest that the primary homeostatic mechanism we observe is a reduction of inhibition onto L2/3 excitatory neurons following sensory deprivation via monocular enucleation.

Our electrophysiology recordings are presumably from a mixed population of recovering and inactive cells. A possibility is that homeostatic mechanisms are engaged exclusively in cells that recover, which may not be apparent in our pooled electrophysiology measurements. Thus, we wanted to separately examine homeostatic mechanisms in cells that recover and those that become inactive. Given that neurons are defined as either recovering or inactive 72 hr after enucleation, we focused on this time point. We hypothesized that we could use immunolabeling to detect the activity/plasticity marker c-Fos (Barth et al., 2004; Yassin et al., 2010) as a proxy for the presence or absence of in vivo activity in a given cell. By immunostaining for c-Fos after electrophysiology recordings of homeostatic mechanisms in acute slices, we would be able to post hoc identify whether the neurons had been active or inactive in vivo and thus classify them as putative recovering or inactive cells.

**Figure 4:**
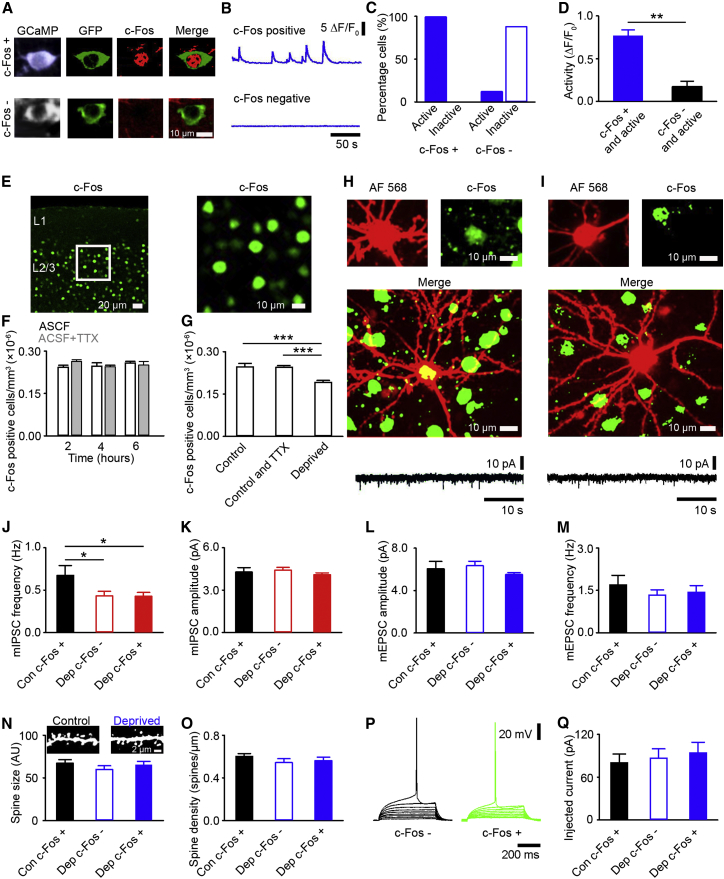
Reduced Inhibition in Putative Recovering and Inactive Neurons (A) Example of GCaMP5 expressing neurons in vivo (left), after sectioning and reconstruction with immunolabeled GFP (middle left), c-Fos (middle right), and merge of GFP and c-Fos images (right) for a c-Fos-positive (top) and -negative (bottom) neuron. (B) Example in vivo GCaMP5 responses from neurons in (A). (C) Percentage of neurons that are: c-Fos positive and active or inactive in vivo and c-Fos negative and active or inactive in vivo (n = 157 reconstructed cells). (D) Average in vivo activity of c-Fos-positive (blue) and c-Fos-negative (black) neurons that are active in vivo (p = 0.003 and t test). (E) The left shows, from a naive mouse, example cells from slices with immunolabeled c-Fos. The right shows a zoom of section from L2/3 denoted by white box in the left image. (F) Density of c-Fos-positive cells in acute visual cortex slices recovered in either artificial cerebrospinal fluid (ACSF) (white) or ACSF and TTX (gray) 2, 4, or 6 hr after slicing. (ACSF versus TTX: 2 hr and p = 0.623; 4 hr and p = 0.981; and 6 hr, p = 0.991, and ANOVA. ACSF: 2 hr and n = 126; 4 hr and n = 128; and 6 hr and n = 134 cells. TTX: 2 hr and n = 137; 4 hr and n = 127; and 6 hr and n = 130 cells). (G) Density of c-Fos-positive cells in acute visual cortex slices that recovered for 4 hr in ACSF or ACSF and TTX from control animals or for 4 hr in ACSF and TTX from animals 72 hr post-enucleation (enucleation [n = 299 cells] versus control TTX [n = 127 cells] and p < 0.001 and enucleation versus control ACSF [n = 128 cells] p < 0.001 and ANOVA with post hoc test). (H and I) Example c-Fos-positive (H) or -negative (I) L2/3 excitatory neuron with electrophysiology recordings. The neurons were filled with AF 568 (top left) and immunostained for c-Fos (top right; merged images, middle). The bottom shows an example of mEPSC recordings from respective cells. (J and K) Average mIPSC frequency (J) and amplitude (K) (control c-Fos positive [black, n = 6] versus enucleation c-Fos negative [white, n = 7]; J, p = 0.034; K, p = 0.895 and control c-Fos positive versus enucleation c-Fos positive [red, n = 5]; J, p = 0.037 and K, p = 0.756 and ANOVA with post hoc test). (L and M) Average mEPSC amplitude (L) and frequency (M) (control c-Fos positive [black, n = 6] versus enucleation c-Fos negative [white, n = 7]: L, p = 0.671; M, p = 0.159 and control c-Fos positive versus enucleation c-Fos positive [blue, n = 6]; L, p = 0.695; M, p = 0.269 and ANOVA with post hoc test). (N and O) Average dendritic spine size (N) and density (O) from control c-Fos-positive (black, n = 112 spines), enucleated c-Fos-negative (white, n = 116 spines), or enucleated c-Fos-positive neurons (blue, n = 109 spines; spine size: p = 0.092 and spine density: p = 0.308 and ANOVA). The inset shows example dendrites from control and enucleated animals. (P) Example voltage response to a series of somatic current injections (0 to 0.1 nA). (Q) Average current threshold for a single AP (control c-Fos positive [black, n = 9] versus enucleation c-Fos negative [white, n = 10] and p = 0.715; control c-Fos positive versus enucleation c-Fos positive [blue, n = 5], p = 0.875, and ANOVA with post hoc test). All recordings are from L2/3 excitatory cells and at 72 hr post-(sham) enucleation (^∗^p < 0.05 and ^∗∗^p < 0.01). The error bars represent SEM. See also Figure S3.

We first tested whether c-Fos reliably reports the activity we observe in vivo. We therefore measured activity levels 72 hr after enucleation in behaving mice expressing GCaMP5. Following the completion of our imaging, we prepared acute slices of visual cortex and allowed these slices to recover for 4 hr to mimic the timing of an electrophysiology experiment. We then immunolabeled for GFP and c-Fos and reconstructed the cells we imaged in vivo. This approach allowed us to identify whether cells that were active in vivo were also c-Fos positive (Figures 4A and 4B). We found that c-Fos immunostaining reflected in vivo activity extremely well. All reconstructed c-Fos-positive cells had been active during the in vivo imaging at 72 hr (Figure 4C), while only 12% of cells that were c-Fos-negative had been active in vivo (Figure 4C). These false negative cells had low levels of GCaMP5 activity in vivo (Figure 4D). Note that although we only measure c-Fos expression and in vivo activity at this single time point (72 hr), in our chronic imaging data, only 3% of cells that were inactive prior to deprivation become active 72 hr after enucleation. Thus, cells that are active in vivo at 72 hr post-enucleation are very likely to be recovering, as per our definition in Figure 2.

To ensure that GCaMP5 expression and the timing of the slicing procedure were not influencing our results, in a separate set of wild-type mice, we prepared acute slices and immunostained for c-Fos (Figure 4E). We quantified the density of c-Fos-positive cells in visual cortex slices and found no density change 2, 4, or 6 hr after the preparation of acute slices (Figure 4F). In slices prepared from mice that had undergone enucleation 72 hr prior, we found a decrease in the density of c-Fos-positive cells (Figure 4G), consistent with our GCaMP5 results. Finally, since synaptic properties are measured in the presence of tetrodotoxin (TTX), we examined the effect of extended exposure of the acute slice to TTX and found this had no effect on the density of c-Fos-positive cells (Figures 4F and 4G).

Having established c-Fos immunohistochemistry as a method for distinguishing between the populations of cells that putatively recover and become inactive in our homeostatic paradigm, we used whole-cell patch clamp electrophysiology to measure homeostatic mechanisms in acute slices prepared from mice that had undergone enucleation 72 hr prior. During these experiments, we filled our recorded cells with Alexa Fluor 568 (AF 568), which allowed for their post hoc identification. Following the completion of our electrophysiology experiments, we immunostained for c-Fos to determine if the cell from which we had recorded was c-Fos positive (putative recovering, Figure 4H) or c-Fos negative (putative inactive, Figure 4I). Further, we used measures of homeostatic plasticity mechanisms that induced limited neuronal activity, as extended synaptic or somatic stimulation could induce c-Fos expression.

We first checked for changes in synaptic inhibition onto the L2/3 excitatory cells by measuring the frequency and amplitude of mIPSCs. We found that, consistent with our averaged results (Figures 3A and 3B), mIPSC frequency (Figure 4J), but not amplitude (Figure 4K), was significantly decreased relative to control in both c-Fos-positive and c-Fos-negative cells, suggesting that there are fewer inhibitory synapses onto both populations of cells after enucleation. This result suggests that the reduction of synaptic inhibition alone does not explain the difference between putative recovering and inactive cells.

We next measured if synaptic scaling is selectively induced in putative recovering cells by comparing mEPSC recordings in c-Fos-positive and c-Fos-negative excitatory neurons. We found no difference in either mEPSC amplitude (Figure 4L) or frequency (Figure 4M) between c-Fos-positive or -negative cells in acute slices prepared from mice that had undergone enucleation 72 hr prior or in cells from control animals. Because our cells were filled with AF 568, we were able to measure the size (integrated brightness) and density of the dendritic spines, as a structural proxy for synaptic scaling (Keck et al., 2013; Wallace and Bear, 2004). We found no differences in either spine size (Figure 4N) or density (Figure 4O) between c-Fos-positive and c-Fos-negative cells 72 hr post-enucleation or in control cells, suggesting that synaptic scaling is not selectively induced in putative recovering cells.

We then examined differences in excitability. Given that extended cellular activity may induce c-Fos expression, we measured excitability via the action potential threshold, the current necessary to evoke a single action potential. We found that the percentage of c-Fos-positive cells during recordings with single action potential induction was similar to the percentage of c-Fos-positive cells during whole-cell recordings without action potential induction (c-Fos positive with single AP induction: 57% and c-Fos-positive with whole-cell recordings without APs: 54%), suggesting that we were not inducing c-Fos expression with these single APs. We found no difference in AP threshold in control cells or c-Fos-positive and -negative cells after enucleation (Figures 4P and 4Q). Furthermore, we measured no difference in passive membrane properties between c-Fos-positive and c-Fos-negative cells (Table 1). Overall, these data suggest that homeostatic mechanisms occur following monocular enucleation, which in L2/3 is primarily a reduction in synaptic inhibition. This reduction is apparent in both putative recovering and inactive cells, suggesting that homeostatic mechanisms are widely expressed. These data are consistent with previous findings suggesting that synaptic inhibition is reduced following sensory deprivation (Chen et al., 2011, 2012; Keck et al., 2011; van Versendaal et al., 2012).

Having measured homeostatic mechanisms that may influence changes in the excitatory cell activity, we next asked if synaptic reorganization might play a role in the homeostatic recovery of activity. We have previously reported that there is structural reorganization of dendritic spines on excitatory cells following sensory deprivation in the adult visual cortex (Keck et al., 2008), which may facilitate functional recovery. Therefore, we examined the dynamics of dendritic spines on L2/3 cells following enucleation using chronic structural imaging in vivo. We calculated the survival fraction, i.e., the fraction of spines that were present prior to enucleation, that are still present at each subsequent time point. This measure is often used as an indication of neuronal circuit stability. We found that the survival fraction was higher after enucleation (Figure S3A), such that a larger number of spines were maintained after deprivation relative to control over the observed time course of homeostatic plasticity (72 hr post-deprivation). While we cannot separate recovering and inactive cells in these experiments, we found no evidence of a bimodal distribution of spine survival fractions 72 hr after enucleation (Figure S3B). In fact, most dendritic branches showed no spine changes (16/20). This increase in synaptic stability suggests that the same presynaptic partners from before deprivation are likely maintained, at least over this short time course, and thus increases in activity are unlikely due to changes in excitatory synaptic connectivity. This result is somewhat surprising, given that sensory deprivation over longer time courses has typically been associated with increased spine dynamics (Grutzendler et al., 2002; Holtmaat et al., 2006; Keck et al., 2008; Trachtenberg et al., 2002; Tropea et al., 2010); however, some forms of sensory deprivation have been shown to promote stability of dendritic spines (Zuo et al., 2005).

### Recovering and Inactive Excitatory Neurons Form Subnetworks

Having found no differences in the individual cell properties between recovering and inactive neurons, we examined if there may be differences between these cells in their local networks. As subnetworks of cells have previously been described in the visual cortex (Ko et al., 2011; Miller et al., 2014; Yoshimura et al., 2005), we investigated the local network interactions of recovering and inactive neurons to determine if local subnetworks of cells play a role in the recovery of activity. During the pre-enucleation time points, we measured the correlation of the GCaMP5 signals between pairs of cells whose activity was imaged simultaneously within a single cortical region. We considered cells to be correlated if this correlation coefficient was significant and positive.

**Figure 5:**
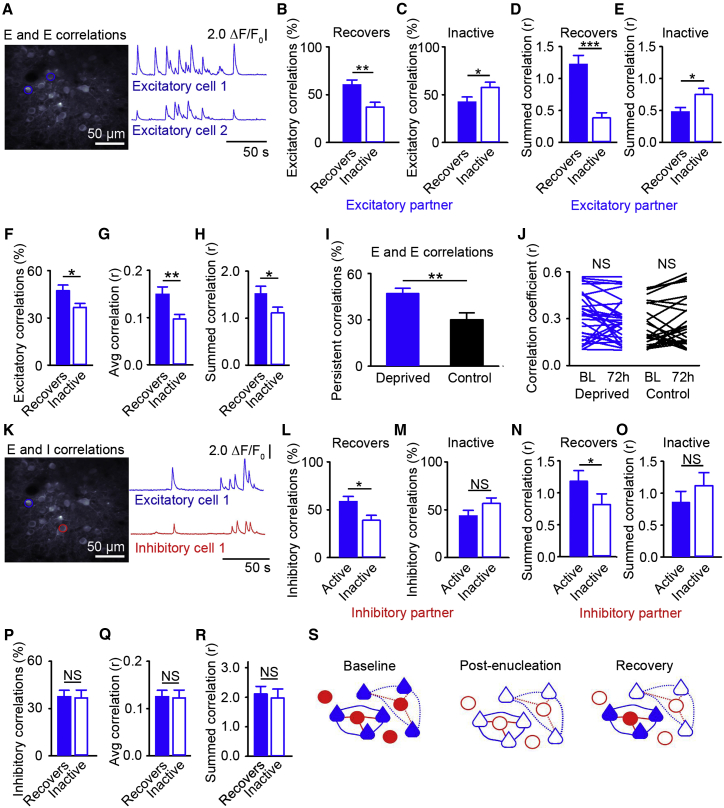
Correlated Excitatory Neurons Recover Cortical Activity (A and K) Correlations between an excitatory neuron with an excitatory (A) or an inhibitory (K) neuron partner. The left shows a maximum intensity projection, with a pair of significantly correlated neurons marked by circles. The correlations were measured in the baseline time points (−24 and −1 hr) prior to enucleation. The right shows ΔF/F_0_ traces from cells in the projection. For these examples, (A) r = 0.48 and p < 0.05; (K) r = 0.144 and p < 0.05. (B and C) Percentage of the total number of recovering (B) or inactive (C) excitatory neuron significant correlations that are with a recovering (filled) or an inactive excitatory partner (open) (B, p = 0.001 and C, p = 0.020 and t test). (D and E) For each recovering (D) or inactive (E) excitatory neuron, the sum of significant correlation coefficients with recovering (filled) or inactive (open) excitatory partners (D, p < 0.001 and E, p = 0.036 and t test). (F and P) For recovering (filled) or inactive (open) excitatory neurons, percentage of the significantly correlated excitatory (F) or inhibitory (P) neurons out of all the excitatory (F) or inhibitory (P) neurons in a region (F, p = 0.024 and P, p = 0.390 and t test). (G and Q) Average correlation coefficient value for significant correlations of recovering (filled) and inactive (open) excitatory neurons with excitatory (G) or inhibitory (Q) partners (G, p = 0.006 and Q, p = 0.911 and t test). (H and R) For each recovering (filled) or inactive (open) excitatory neuron, the sum of the significant correlation coefficients with excitatory (H) or inhibitory (R) partners (H, p = 0.037 and R, p = 0.870 and t test). (I) For recovering excitatory neurons with excitatory neuron partners, percentage of the total number of cells that were significantly correlated prior to enucleation (blue) or control (black) that remain correlated 72 hr later (p = 0.005 and t test). (J) Correlation coefficients during baseline (BL) and 72 hr after (sham) enucleation for pairs of persistently correlated excitatory neurons. Each line is a pair of neurons (enucleation: BL versus 72 hr and p = 0.383 and control: BL versus 72 hr, p = 0.088, and paired t test). (L and M) Percentage of the total number of recovering (L) or inactive (M) excitatory neuron significant correlations that are with either an active (filled) or an inactive (open) inhibitory partner (L, p = 0.018 and M, p = 0.106 and t test). (N and O) For each recovering (N) or inactive (O) excitatory neuron, the sum of significant correlation coefficients with active (filled) or inactive (open) inhibitory partners (N, p = 0.030 and O, p = 0.387 and t test). (S) Schematic of subnetworks of excitatory (blue) and inhibitory (red) neurons. The lines indicate correlations prior to deprivation for recovering (solid) and inactive (dashed) subnetworks (^∗^p < 0.05, ^∗∗^p < 0.01, ^∗∗∗^p < 0.001, no significance [NS], and the error bars represent SEM). See also Figure S4.

We first examined correlations between putative excitatory neurons (Figure 5A), which likely reflect either preferential connections between cells and/or common inputs (Hofer et al., 2011; Ko et al., 2011; Komiyama et al., 2010; Miller et al., 2014; Yoshimura et al., 2005), as recurrently connected cells are likely to share common inputs in rodent visual cortex. We found that prior to deprivation, excitatory neurons that would later undergo homeostatic recovery were more likely to be correlated with other recovering excitatory neurons (Figure 5B). Specifically, when the significant correlation values (r) for each recovering excitatory cell were summed (a measure for the combined number and strength of correlations), a majority of the correlation for cells that would recover was with other recovering cells (Figure 5D). Conversely, cells that would become inactive were more often correlated with other cells that would become inactive (Figure 5C) and when the significant correlation values were summed, a majority of the correlation for inactive cells was with other cells that would become inactive (Figure 5E). These results suggest that recovering and inactive neurons form subnetworks of cells.

It is important to note that these two groups of correlated cells are not completely distinct and there are some significant correlations between those cells that will recover and those that become inactive (Figures 5B–5E). Further, not all recovering cells within a region have significant correlations with one another (Figures 5B–5E). When we further examined the properties of the recovering and inactive cells in their subnetworks, we found that recovering neurons had a higher number of significant correlations (i.e., more cells in their subnetwork, Figure 5F) and that these correlations on average were stronger (Figure 5G), such that when the total significant correlations for individual recovering cells were summed, they had a higher total correlation value than measured for neurons that would become inactive (Figure 5H). These results suggest that the recovering neurons are embedded in larger and more strongly correlated subnetworks prior to deprivation.

As higher cellular activity levels have been demonstrated to give rise to increased correlations (de la Rocha et al., 2007), we quantified cellular activity levels before enucleation and found no difference between recovering and inactive cells (Figure S4A), as well as no correlation between the baseline cellular activity level and the number of correlated cells or correlation strength (Figures S4B–S4D). Furthermore, to ensure that we did not have a sampling bias based on spatial organization, we looked at the spatial relationship between recovering and inactive excitatory neurons and found them not to spatially cluster in any apparent way (Figure S4E). To determine if our c-Fos reconstructions reflect these subnetworks of putative recovering cells, we examined the in vivo correlations for c-Fos reconstructed cells 72 hr post-enucleation (as in Figure 4). We found that all c-Fos-positive cells had correlated activity in vivo with at least one other c-Fos positive cell, and that on average, a c-Fos-positive cell was correlated with 73% ± 3% of the other c-Fos-positive cells in an imaging region, consistent with previous work (Yassin et al., 2010). Finally, to examine if there are common inputs that are driving the recovering subnetwork, we measured the correlation coefficients of the inactive and recovering cells during different behavioral periods in our paradigm to determine if these subnetworks were active together during particular behavioral stimuli (thus implying a common input). We found that the normalized correlation for recovering and inactive subnetworks prior to deprivation was the same during each of our behavioral measures (Figure S4F). These data indicate that there is not a specific set of behavioral inputs that are predominantly driving either the recovering or inactive subnetwork prior to deprivation.

### Subnetworks of Recovering Cells Are Maintained after Deprivation

We next asked if these subnetworks are maintained over time, specifically during recovery. We examined the fraction of cells that remain correlated at 72 hr, out of all cells that were correlated prior to enucleation. We found that in control animals the subnetworks were highly dynamic, consistent with previous studies of subnetworks in naive animals (Miller et al., 2014). Only 30% of cells remained correlated after 72 hr (Figure 5I). Conversely, we found that following sensory deprivation, subnetworks were more likely to be maintained, as 52% of the recovering subnetwork was persistent and remained correlated 72 hr after enucleation (Figure 5I). Across time, the strength of persistent correlations between excitatory neurons remained stable in both enucleated and control animals (Figure 5J). These results are consistent with an increase in synaptic stability (Figure S3), which taken together suggest an overall increase in network stability in the 72 hr following enucleation.

We then examined correlations in the persistent subnetwork in each behavioral condition to examine if common inputs or behaviors are driving the persistent subnetwork. Consistent with an increase in spontaneous activity in individual recovering cells, we found that relative to pre-enucleation time points, the correlation coefficient for the persistent recovering subnetwork increased 72 hr after enucleation when the mouse was stationary in the dark (our proxy for spontaneous activity), but in no other behavioral conditions (Figure S4G). These data suggest that spontaneous activity may play a role in facilitating homeostatic recovery.

### Recovering Subnetworks Include Excitatory and Inhibitory Neurons

We next measured pairwise correlations in activity prior to deprivation between the excitatory neurons and their neighboring inhibitory neurons to examine the role of inhibitory cell activity in the subnetworks (Figure 5K). When we examined correlations between excitatory cells and either active or inactive inhibitory cell partners during baseline activity, we found that recovering excitatory cells were more likely to be correlated with inhibitory partners that maintain their activity after enucleation rather than with inhibitory partners that become inactive (Figures 5L and 5M). Further, the sum of the significant correlations for excitatory neurons that will recover is greater with inhibitory partners that will remain active (Figures 5N and 5O). We also found that excitatory neurons that recovered their activity were correlated with a similar number of local inhibitory partners as those excitatory neurons that became inactive (Figure 5P) and that the strengths of these correlations with inhibitory partners were not different between cells that would recover or become inactive (Figures 5Q and 5R), indicating that the recovering and inactive subnetworks have similar levels of correlated inhibition prior to deprivation. Together, these results suggest that there are subnetworks of excitatory neurons and associated inhibitory cells that are correlated prior to deprivation and these subnetworks either recover their activity or become inactive following sensory deprivation (Figure 5S).

## Discussion

Here, we show that a subset of excitatory neurons homeostatically recover activity levels following sensory deprivation. Prior to deprivation, these recovering cells are: (1) more strongly correlated with a larger number of cells and (2) preferentially correlated with other cells that will also recover. Furthermore, the recovering excitatory cells are more often correlated with inhibitory cells that remain active following deprivation, indicating that there is a subnetwork of excitatory and inhibitory cells involved in the homeostatic recovery of activity observed in our experimental paradigm. We measure a shift in the balance of synaptic excitation and inhibition, toward reduced synaptic inhibition, in both recovering and inactive cells. Together these results implicate a role for a previously described homeostatic mechanism—decreased inhibition—and the local network in the homeostatic recovery of activity following sensory deprivation.

### Mechanisms of Homeostatic Plasticity

Previous work (Hengen et al., 2013; Keck et al., 2013) has demonstrated homeostatic recovery in the average activity across all cells in the population. The degree of change in average activity that we measure here (Figure S2A) is consistent with our previous work using GCaMP5, where following binocular deprivation via retinal lesions (as opposed to monocular enucleation used here), the decrease in activity was greater and subsequent recovery was less than that observed with GCaMP3 (Keck et al., 2013). This difference is likely due to the relative sensitivities of the two indicators (Akerboom et al., 2012). Furthermore, both of these previous studies (Hengen et al., 2013; Keck et al., 2013), despite using different paradigms and ages (monocular deprivation in juvenile and binocular lesions in adult mice, respectively), demonstrated synaptic scaling of excitatory synapses, which we do not observe here. Many different factors influence the homeostatic plasticity mechanisms that are expressed in the cortex. First, varying deprivation paradigms, such as monocular deprivation with eyelid suture or monocular enucleation, which presumably result in differences in activity patterns and/or changing activity levels, have been demonstrated to induce different mechanisms of homeostatic plasticity (Maffei and Turrigiano, 2008). Second, there is a developmental time course to homeostatic mechanisms, expressed in a layer specific manner (Desai et al., 2002; Goel and Lee, 2007; but see Lambo and Turrigiano, 2013). This layer specificity may play a role in adult homeostatic plasticity as well. Synaptic scaling has previously been observed in L5, as has a shift in the E/I balance following binocular deprivation with retinal lesions (Keck et al., 2013). Here, in L2/3 using monocular enucleation, we did not observe synaptic scaling, but did find a shift in the E/I balance, suggesting that reduced synaptic inhibition influences cells in both L2/3 and L5 and following different deprivation paradigms. These results are consistent with mounting evidence across cell layers (both L2/3 and L5) and deprivation paradigms (monocular deprivation, monocular enucleation, and focal and binocular retinal lesions) that a reduction of synaptic inhibition precedes excitatory plasticity in adulthood and therefore may be permissive for reorganization (Chen et al., 2011, 2012; Keck et al., 2011, 2013; van Versendaal et al., 2012).

### Implications of Subnetworks for Homeostatic Plasticity

We have found that excitatory cells that are correlated with larger networks of recovering cells are also more likely to recover their activity following deprivation, which is likely facilitated by the reduction of synaptic inhibition. Given, however, that we see reduced synaptic inhibition in both putative recovering and inactive neurons, our data suggest that the local network may also play a role in homeostatic recovery. An interpretation of these data is that homeostatic mechanisms facilitate recovery, but that excitatory cell activity is necessary for this recovery, either activity via recurrent connections or common inputs. Thus, only cells that are part of a sufficiently extensive subnetwork have enough maintained drive to recover their activity.

Given our data, we cannot distinguish between the subnetworks having common inputs, recurrent connections, or some combination of the two, but any of these three possibilities could provide the neural drive necessary to homeostatically restore cortical activity. Previous work has indicated that excitatory cells with correlated activity are interconnected with a high probability (Ko et al., 2011) and that reciprocally connected cells are highly likely to share common inputs (Yoshimura et al., 2005). Thus, our correlated cells (both recovering and inactive) are likely to be connected, as well as having shared inputs.

If common inputs are involved in the recovery of activity, what might these inputs be? From the data presented here, these inputs are unlikely to be direct visual inputs, as we have removed the principal visual drive into the monocular visual cortex and no longer observe visual stimulus-locked responses after enucleation (Figures S2D and S2E). Preserved inputs could come from a number of other brain regions, as evidence for auditory (Iurilli et al., 2012) and motor (Andermann et al., 2011; Ayaz et al., 2013; Keller et al., 2012; Niell and Stryker, 2010; Saleem et al., 2013) related input has been demonstrated in mouse visual cortex. Additionally, maintained inputs could arise from internal state, which has previously been implicated in subnetworks in mouse visual cortex (Miller et al., 2014) or contextual feedback from higher areas (Tohmi et al., 2014; Yoshitake et al., 2013), which could be driven by the spared eye via higher visual areas. Given that we observe an increase in the stability of dendritic spines in deprived mice over this short time course (Figure S3), we believe that it is unlikely that pre-existing inputs are removed and new synaptic connections are formed during this time, as new connections would be associated with increased spine dynamics, as has been previously described over a longer time course (Grutzendler et al., 2002; Holtmaat et al., 2006; Keck et al., 2008; Trachtenberg et al., 2002; Tropea et al., 2010). Finally, if the correlations observed prior to deprivation reflect common inputs, one possible explanation for the inactive subnetwork, which was visually responsive prior to deprivation, is that the common input that drives that inactive subnetwork after deprivation is not stimulated in our experimental paradigm. This could explain why these cells become unresponsive and would further implicate the importance of network inputs in homeostatic recovery.

Additionally, correlated cells may be preferentially connected to one another or their connections may have a greater synaptic efficacy (Cossell et al., 2015). Consistent with our result of spontaneous activity increasing over the time course of recovery (Figures S2E and S4F), synaptically connected networks of cells are capable of amplifying spontaneous activity, particularly in mixed networks of excitatory and inhibitory cells, where maintained inhibitory cell activity is thought to stabilize the network (Murphy and Miller, 2009). This amplification of network activity could, in part, explain our observed homeostatic recovery of the larger subnetwork, and provide an indication of why inhibitory cells are more likely to remain active in recovering subnetworks. Most likely, some combination of synaptic connections and shared inputs drive the activity measured in the subnetwork of recovering excitatory cells (Yoshimura et al., 2005) and this activity is further increased by the reduction of synaptic inhibition measured in these cells. Thus, a combination of network properties and homeostatic mechanisms facilitate functional recovery in the visual cortex.

### Plasticity of Inhibitory Cells

A large fraction of the measured inhibitory neurons become inactive after sensory deprivation. In the inhibitory neurons that remain active, we do not observe a strong homeostatic recovery, in contrast to previous reports in developing animals (Hengen et al., 2013). A possible explanation for these differences is the developmental stage, as inhibition, as well as the visual cortex in general, is known to be more plastic during the critical period than after (Hensch, 2004). Another possibility is that the in vivo extracellular electrophysiology experiments of Hengen et al. (2013) focused on fast-spiking inhibitory cells, whereas our immunohistochemical approach includes a range of inhibitory cell-types (see Experimental Procedures). Given the differences in synaptic targets of inhibitory cell types (Pfeffer et al., 2013), they may play different roles in homeostatic recovery and thus could have different recovery profiles. Specifically, the difference between active and inactive inhibitory cells in our data could simply be a subtype specific effect. Further work is necessary to understand inhibitory cell-type specific responses to sensory deprivation and their relative roles in homeostatic plasticity.

Why might the inhibitory cells correlated with the recovering subnetwork remain active? Either common excitatory inputs to or strong connectivity between the excitatory and inhibitory cells may drive the inhibitory cell activity observed after deprivation. Thus, while synaptic inhibition is widely decreased to facilitate cortical plasticity following sensory deprivation (Chen et al., 2011), only the inhibitory cells associated with the recovering excitatory cells remain active. This subnetwork specific inhibitory cell activity could maintain network stability in recovering subnetworks by preventing unchecked excitatory activity. These results are consistent with theoretical work (Murphy and Miller, 2009) that shows that some level of inhibitory cell activity is necessary to maintain stable network activity.

Finally, following sensory deprivation, we also observe an increased stability in the correlations of the subnetwork of cells that are correlated prior to deprivation, as well as an increase in the stability of the dendritic spines. Similar increases in stability of neural ensembles and associated spine dynamics have been observed to develop during motor learning (Peters et al., 2014). Thus, while underlying mechanisms may differ between learning and deprivation, the increase in stability of the active excitatory subnetwork we observe here may reflect a general principle of cortical plasticity common to both.

## Experimental Procedures

For a complete description of the methods, see the Supplemental Information.

### Animals and Surgery

All experiments were conducted according to the United Kingdom Animals (Scientific Procedures) Act 1986 or were approved by the Veterinary Department of the Canton of Basel-Stadt, Switzerland. We used age and sex matched adult (P58–P200) male and female C57BL/6 mice or in a subset of experiments, GFP-M (Feng et al., 2000) mice. Cranial windows were surgically implanted over the monocular primary visual cortex in ketamine-xylazine anesthetized mice (Holtmaat et al., 2009). For experiments involving GCaMP5, mice were injected with AAV2/1-*ef1*α-GCaMP5 during the craniotomy. Following surgeries, mice recovered for at least 18 days before experiments. Monocular enucleation involved lidocaine application prior to surgical removal of the eye.

### Functional and Structural Imaging

Structural and functional imaging experiments were performed as described previously (Keck et al., 2013). Briefly, imaging was performed with a two-photon laser-scanning microscope. In functional imaging experiments, a high power objective Z-Piezo stage was used to acquire volume measurements. Awake experiments consisted of alternating 3 min blocks in which the mouse either received coupled visual feedback—full field gratings controlled by the movement of the mouse—or the screens were off. This was followed by drifting gratings in eight directions (0–360 degrees by 45 degree steps) presented in a random order, with a spatial frequency of 0.04 cycles/degree and a temporal frequency of 2 Hz. The same visual stimulus paradigm was used at each time point before and after enucleation.

### Data Analysis

All structural data were analyzed blind to the experimental condition, as previously described (Keck et al., 2013). Functional imaging data were full-frame registered and regions of interest were chosen by hand from high-resolution depth stacks (Keck et al., 2013). Neurons were considered visually responsive if they had significantly more activity during visual stimulation than in darkness, in the absence of locomotion.

After in vivo imaging, we sectioned the brains. Using fluorescent beads injected in vivo for guidance, we reconstructed the neurons previously functionally imaged in vivo and performed immunohistochemistry to identify them as positive or negative for either GABA or c-Fos (Kerlin et al., 2010), blind to the in vivo activity profiles. Neurons were considered to be GABA/c-Fos positive if somatic labeling was 20% greater than background levels. Following reconstructions with GABA immunostaining, we used calcium transient kinetics of inhibitory and excitatory neurons to train a regression tree classification algorithm on a randomly selected half of the data set and then cross-validated it on the other half of the data set, which was novel to the classifier. Performance on this novel data set was 91% correct.

We computed the pairwise correlations between calcium traces of all active neurons in the same cortical region and included cells with positive and statistically significant (p < 0.05) correlation coefficients in our analyses. Persistent correlations were significant both in baseline and 72 hr after (sham) enucleation.

### Electrophysiology

Acute slices were prepared from the visual cortex of adult mice following enucleation or sham-enucleation and whole cell patch clamp recordings were made from L2/3 cells in the monocular visual cortex, as described previously (Keck et al., 2013).

### Statistics

Comparisons were made using parametric (t test, ANOVA with Holm-Sidak post hoc test, repeated-measures ANOVA with post hoc test) or non-parametric (Mann-Whitney rank sum, ANOVA on ranks) statistics where appropriate. Correlation coefficients were calculated with a Spearman’s rank correlation coefficient.

## Figures and Tables

**Table 1:** Enucleation Does Not Modify Resting Membrane Potential Properties

Passive Membrane Properties	Time Points			
	Control (n = 22)	24 hr (n = 13)	48 hr (n = 12)	72 hr (n = 16)
Input resistance (MΩ)	140 ± 20	130 ± 10	120 ± 9	150 ± 13
Membrane time constant (ms)	31 ± 3	27 ± 3	27 ± 2	32 ± 2
Membrane capacitance (pF)	220 ± 8	210 ± 10	230 ± 10	220 ± 5

**Passive membrane properties**	**c-Fos Positive (n = 18)**		**c-Fos Negative (n = 12)**	

Input resistance (MΩ)	170 ± 20		150 ± 20	
Membrane time constant (ms)	37 ± 7		27 ± 3	
Membrane capacitance (pF)	210 ± 20		180 ± 10	

Enucleation did not modify input resistance (p = 0.517 and ANOVA), membrane time constant (p = 0.612 and ANOVA), or membrane capacitance (p = 0.490 and *ANOVA*). The input resistance (p = 0.460 and t test), membrane time constant (p = 0.353 and t test), and membrane capacitance (p = 0.310 and t test) were not different between c-Fos positive and c-Fos negative L2/3 excitatory neurons 72 hr post-enucleation.

## Supplemental Information

Document S1. Supplemental Experimental Procedures and Figures S1–S4

Document S2. Article plus Supplemental Information
