# Supplementary material for: Subnetwork-Specific Homeostatic Plasticity in Mouse Visual Cortex In Vivo
Source: Neuron. 2015 Jun 3;86(5):1290–303. doi: 10.1016/j.neuron.2015.05.010 (PMC4460189; doi:10.1016/j.neuron.2015.05.010)
Supplement: Document S1. Supplemental Experimental Procedures and Figures S1–S4 [file mmc1.pdf]

Neuron

Supplemental Information

Subnetwork-Specific Homeostatic Plasticity in Mouse Visual Cortex In Vivo

Samuel J. Barnes, Rosanna P. Sammons, R. Irene Jacobsen, Jennifer Mackie, Georg B. Keller, and Tara Keck

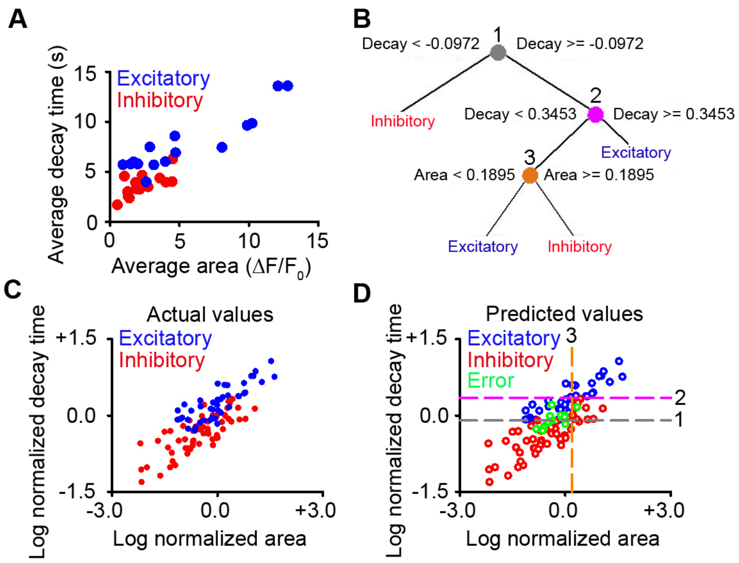

Figure S1, related to Figure 1 – Classifier performance

A) Example data from a cortical region of a single animal showing the predictor values used in cell-type classification (area and decay time). Each point is the average value from a putative excitatory (blue) or putative inhibitory (red) neuron. **B)** Classifier structure with values at decision points for decay time and area (decision point 1, grey; decision point 2, pink; decision point 3, orange). **C)** Log-normalized values of area and decay time, taken from identified putative inhibitory (red) and putative excitatory (blue) neurons. **D)** Same data as in Panel C, but color-coded (blue excitatory, red inhibitory, green errors) as classified by the model in Panel B. Decision points are shown as dashed lines (colors and numbers are the same as in Panel B). We trained the classification tree on a randomly selected half of the dataset and then cross-validated it on the other half of the dataset, which was novel to the classifier.

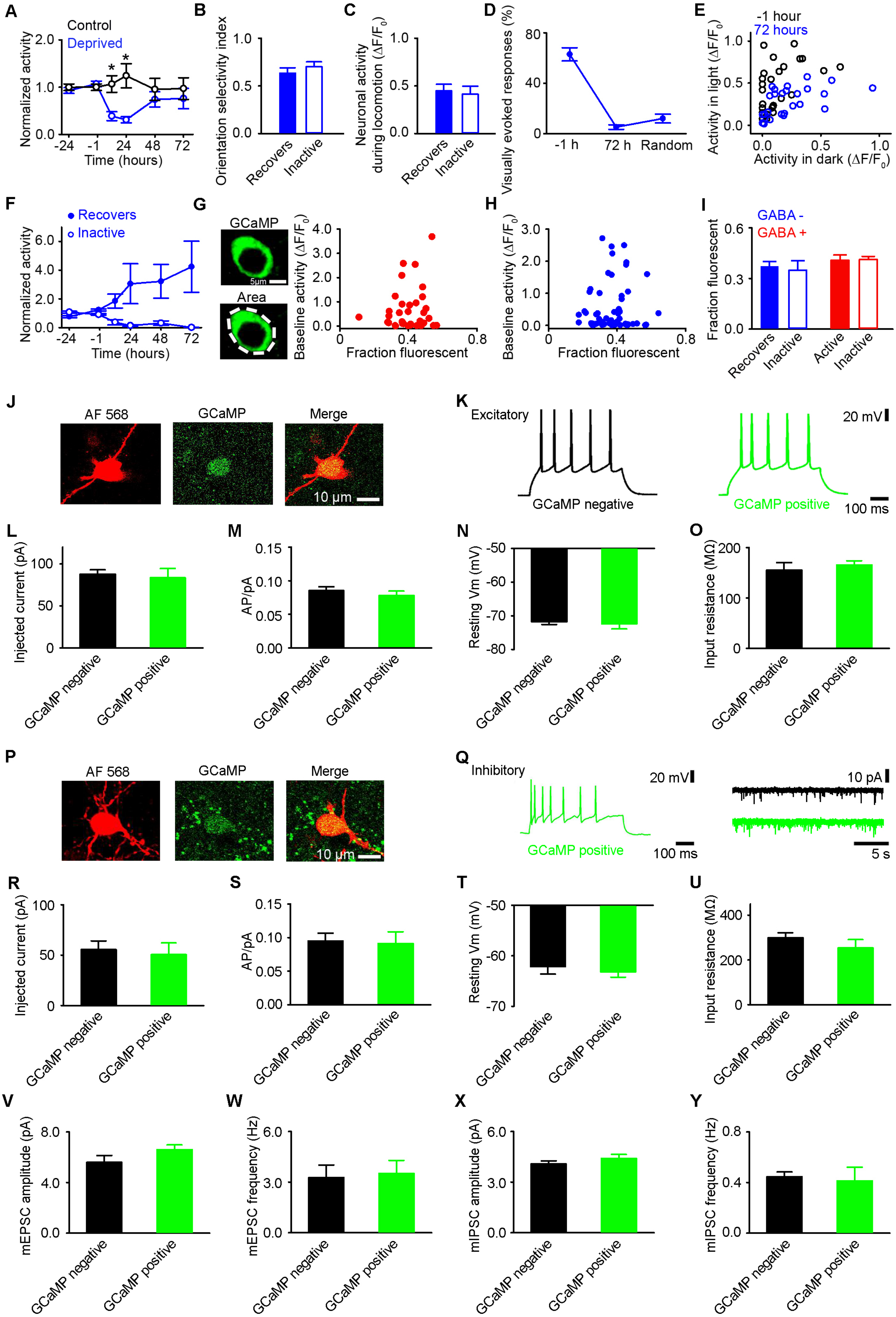
A
Control
Deprived
Normalized activity
Time (hours)
B
Orientation selectivity index
Recovers
Inactive
C
Neuronal activity during locomotion (ΔF/F0)
D
Visually evoked responses (%)
-1 h
72 h
Random
E
-1 hour
72 hours
Activity in light (ΔF/F0)
Activity in dark (ΔF/F0)
F
Recovers
Inactive
G
GCaMP
Area
5μm
Baseline activity (ΔF/F0)
Fraction fluorescent
H
I
GABA -
GABA +
Fraction fluorescent
Active
J
AF 568
GCaMP
Merge
10 μm
K
Excitatory
GCaMP negative
GCaMP positive
20 mV
100 ms
L
Injected current (pA)
M
AP/pA
N
Resting Vm (mV)
O
Input resistance (MΩ)
P
Q
Inhibitory
10 pA
5 s
R
S
T
U
V
mEPSC amplitude (pA)
W
mEPSC frequency (Hz)
X
mIPSC amplitude (pA)
Y
mIPSC frequency (Hz)

Figure S2, related to Figure 2 – Properties of recovering and inactive cells, GCaMP5 expression

A) Activity of all excitatory neurons following enucleation (blue) or in control (black), normalized on a cell-by-cell basis to average activity measured prior to enucleation or sham-enucleation (Control versus enucleation: 12 hr, $p = 0.027$; 24 hr, $p = 0.023$; 48 hr, $p = 0.632$; 72 hr, $p = 0.814$, *t-test*). **B)** Orientation selectivity index for recovering and inactive excitatory neurons (Recovering versus inactive, $p = 0.233$, *t-test*). **C)** Total activity for recovering and inactive excitatory neurons during locomotion (Recovering versus inactive, $p = 0.750$, *t-test*). **D)** Percentage of responses that are time locked to the onset of drifting gratings prior to enucleation (-1 hr) or following enucleation (72 hr), compared with random onsets, calculated by superimposing the timing of the grating stimuli on periods recorded in the dark prior to enucleation (Random) (-1 hr versus 72 hr: $p < 0.001$; Random versus 72 hr: $p = 0.275$, *ANOVA with post-hoc test*). **E)** Activity for individual cells in the dark versus activity in the light, where each point is an individual cell, prior to (black) and 72 hr after enucleation (blue). Average difference between total light and dark responses before enucleation (0.32 ± 0.05) versus after enucleation (0.04 ± 0.03; $p < 0.001$, *t-test*). **F)** Average spontaneous activity (recorded while the animal was stationary in the dark) of recovering (filled) and inactive (open) excitatory neurons (Recovering 72 hr versus inactive 72 hr, $p = 0.020$, *t-test*). **G,H)** Left, example of a GCaMP5 expressing neuron after sectioning and reconstruction, with immunolabeling of GFP (top left). The area of the cell that contained fluorescent pixels above background fluorescence was normalized to the total area of the cell (bottom left). Scale bar, 5 μm. Right, fraction of the neuron that is fluorescent versus total baseline activity for inhibitory neurons ($r = -0.01$, $p = 0.991$, *Spearman's rank correlation coefficient*) and **H)** excitatory

neurons (r = 0.01, p = 0.930, *Spearman's rank correlation coefficient*). **I)** Average fraction of the neuron that is fluorescent for GABA negative recovering (blue filled) and inactive (blue open) neurons (GABA negative: Recovering versus inactive, p = 0.841, *t-test*) and GABA positive active (red filled) and inactive (red open) neurons (GABA positive: Active versus inactive, p = 0.915, *t-test*). **J,P)** Example of a GCaMP5 positive excitatory (J) or inhibitory (P) neuron from which electrophysiology recordings were made. Neurons were filled with AF 568 (left) and then labeled with an antibody against GFP (middle), merged images (right) were used to confirm the presence of GCaMP5. Scale bar, 10μm. **K)** Example action potentials from a GCaMP5 negative (left) or positive (right) excitatory neuron. Scale bar, 20 mV, 100 ms. **L,M,N,O)** For GCaMP5 negative (black, n = 16) and GCaMP5 positive (green, n = 7) excitatory neurons, average action potential current threshold (L), average number of action potentials per picoAmpere (pA) of current injected (M), average resting membrane potential (N), and average input resistance (O) (GCaMP5 positive versus GCaMP5 negative: Panel L, p = 0.701; Panel M, p = 0.429; Panel N, p = 0.678; Panel O, p = 0.673, *t-test*). **Q)** Left, example of multiple action potentials from a GCaMP5 positive inhibitory neuron. Scale bar, 20 mV, 100 ms. Right, example mEPSC traces from GCaMP5 negative (black) and positive (green) inhibitory neurons. Scale bar, 10 pA, 5 seconds. **R,S,T,U)** For GCaMP5 negative (black, n = 11) and GCaMP5 positive (green, n = 10) inhibitory neurons, average action potential current threshold (R), average number of action potentials per picoAmpere (pA) of current injected (S), average resting membrane potential (T), and average input resistance (U) (GCaMP5 positive versus GCaMP5 negative: Panel R, p = 0.724; Panel S, p = 0.842; Panel T, p = 0.450; Panel U, p = 0.297, *t-test*). **V,W)** Average mEPSC amplitude (V) and frequency (W) from GCaMP5 negative (black)

and GCaMP5 positive (green) inhibitory neurons 72 hr after enucleation. (GCaMP5 positive (n = 6) versus GCaMP5 negative (n = 6): Panel V, p = 0.162; Panel W, p = 0.824, *t-test*). **X,Y)** Average mIPSC amplitude (X) and frequency (Y) from GCaMP5 negative (black) and GCaMP5 positive (green) inhibitory neurons 72 hr after enucleation (GCaMP5 positive (n = 6) versus GCaMP5 negative (n = 6): Panel X, p = 0.310; Panel Y, p = 0.788, *t-test*). Panels J-Y are in animals 72 hr post-enucleation.

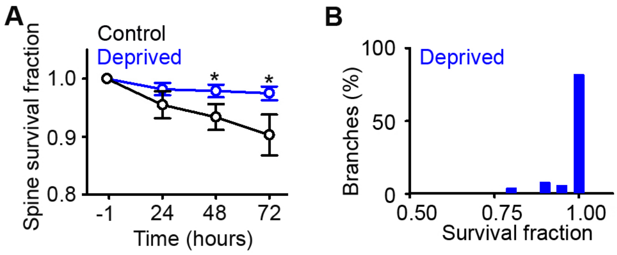

Figure S3, related to Figure 4 - Dendritic spine stability increases after deprivation

A) The survival fraction of dendritic spines on L2/3 excitatory neurons, calculated as the fraction of spines that were present immediately prior to (sham) enucleation (-1 hr) that are still present at each subsequent time point, following enucleation (blue) or in control (black). (Control versus enucleation: 24 hr, $p = 0.243$; 48 hr, $p = 0.049$; 72 hr, $p = 0.027$, *t-test*; Enucleation, $n = 239$ stable spines; Control, $n = 82$ stable spines). **B)** Distribution of dendritic spine survival fraction scores for individual branches 72 hr after enucleation. *$p < 0.05$. Error bars, S.E.M.

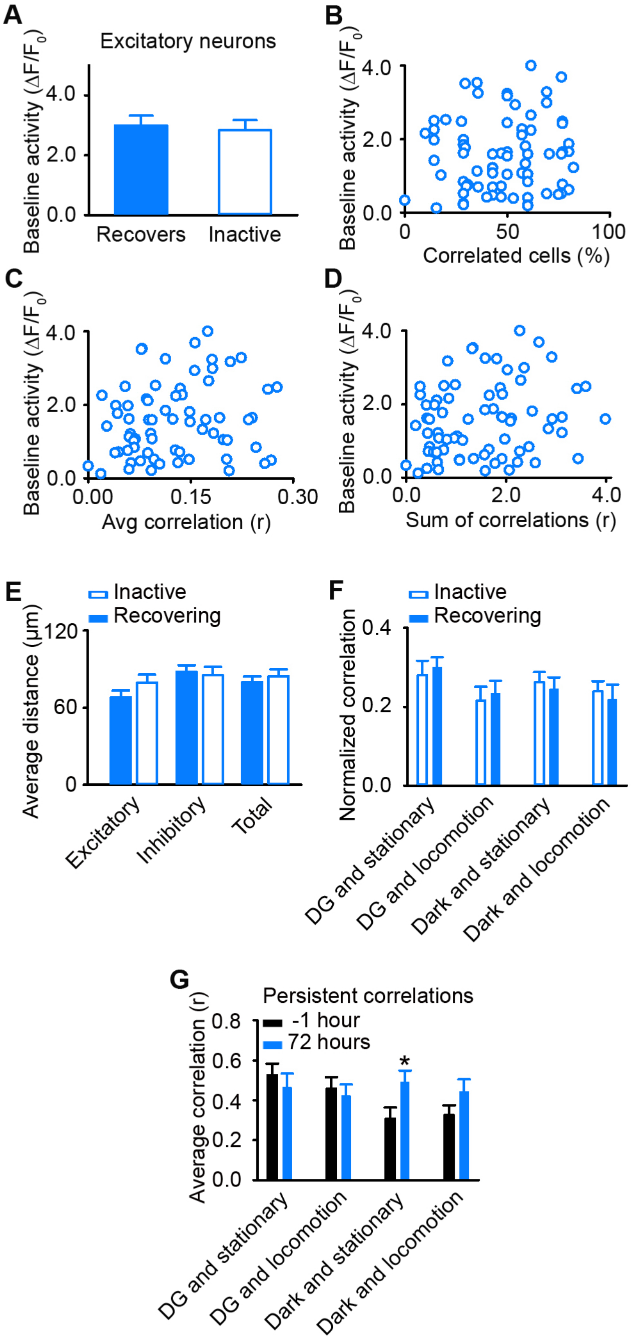
A
Excitatory neurons
Baseline activity (ΔF/F0)
4.0
2.0
0.0
Recovers
Inactive
B
Baseline activity (ΔF/F0)
4.0
2.0
0.0
0
50
100
Correlated cells (%)
C
Baseline activity (ΔF/F0)
4.0
2.0
0.0
0.00
0.15
0.30
Avg correlation (r)
D
Baseline activity (ΔF/F0)
4.0
2.0
0.0
0.0
2.0
4.0
Sum of correlations (r)
E
Inactive
Recovering
Average distance (μm)
120
60
0
Excitatory
Inhibitory
Total
F
Inactive
Recovering
Normalized correlation
0.4
0.2
0.0
DG and stationary
DG and locomotion
Dark and stationary
Dark and locomotion
G
Persistent correlations
-1 hour
72 hours
Average correlation (r)
0.8
0.6
0.4
0.2
0.0
*
DG and stationary
DG and locomotion
Dark and stationary
Dark and locomotion

Figure S4, related to Figure 5 – Baseline activity, spatial distributions and common inputs are similar for inactive and recovering neurons and subnetworks

A) Activity during baseline (average of activity at -24 and -1 hr) over all conditions (visual stimulation and darkness) for excitatory recovering (filled) and inactive (open) neurons (Recovering versus inactive, p = 0.749, *t-test*). **B,C,D)** For each excitatory cell, baseline activity (average of activity at -24 and -1 hr) versus (B) percentage of significant correlations with excitatory cell partners for that cell (r = 0.08, p = 0.486, *Spearman's rank correlation coefficient*), (C) average correlation coefficient of all significant correlations with excitatory cell partners for that cell (r = 0.05, p = 0.654, *Spearman's rank correlation coefficient*), and (D) summed correlation coefficients for all significantly correlated excitatory cell partners for that cell (r = 0.20, p = 0.095, *Spearman's rank correlation coefficient*). **E)** Average distance of recovering (filled) or inactive (open) excitatory neurons to their correlated excitatory partners (Recovering versus inactive, p = 0.151, *t-test*), their correlated inhibitory partners (Recovering versus inactive, p = 0.749, *t-test*) and the combination of both excitatory and inhibitory partners (Recovering versus inactive, p = 0.506, *t-test*). **F)** Average correlation coefficient for recovering and inactive correlations measured in each behavioral condition normalized to the sum of the correlation coefficient in all behavioral conditions (to account for differences in average correlation coefficient in recovering and inactive cells). The normalized correlation coefficient was measured prior to deprivation (-24 and -1 hr) for recovering (filled) and inactive (open) correlations during behavioral conditions: drifting gratings (DG) viewed when the animal is stationary (Recovering versus inactive, p = 0.638, *t-test*), DG during locomotion (Recovering versus inactive, p = 0.350, *t-test*), when the animal is stationary in the dark (Recovering versus inactive, p = 0.652, *t-test*) and during

locomotion in the dark (Recovering versus inactive, $p = 0.666$, *t-test*). **G)** Average correlation coefficient for persistent correlations prior to (black) and 72 hr after enucleation (blue) for each behavioral condition: drifting gratings (DG) viewed when the animal is stationary (-1 versus 72 hr, $p = 0.976$), DG during locomotion (-1 versus 72 hr, $p = 0.965$), when the animal is stationary in the dark (-1 versus 72 hr, $p = 0.025$) and during locomotion in the dark (-1 versus 72 hr, $p = 0.486$, $n = 31$, *repeated measures ANOVA with post-hoc test).* *$p < 0.05$. Error bars, S.E.M.

Supplementary experimental procedures

Animals

All experiments were conducted according to the United Kingdom Animals (Scientific Procedures) Act 1986 or were approved by the Veterinary Department of the Canton of Basel-Stadt, Switzerland. Adult (P58 – 200) male and female mice were used for all experiments. All experimental controls were age and sex matched within experimental groups. Mice were housed in groups of four to five littermates in standard cages and kept on a 12 hour light-dark cycle. 16 (female, ages P58-P120 age matched) C57BL/6 mice were used for functional imaging experiments. 14 (male and female mixed and sex matched, ages P90-140 age matched) mice expressing enhanced green fluorescent protein (GFP) under the *thy-1* promoter (GFP-M line, Feng et al., 2000) were used for structural imaging experiments. Electrophysiology experiments were conducted using C57BL/6 mice (Charles River, UK, male and female mixed and sex matched, ages P60-200 age matched).

Surgery

Cranial windows were surgically implanted over the right hemisphere of monocular primary visual cortex, according to previously described methods (Holtmaat et al., 2009). Briefly, mice were administered with a pre-surgery analgesic (buprenorphine s.c. 0.1 μg/g of body weight) and then anesthetized with an i.p. injection of ketamine (0.15 mg/g of body weight) and xylazine (0.015 mg/g of body weight). A craniotomy was made over monocular visual cortex. A glass cover slip was positioned over the cortex and sealed to the surrounding bone with dental cement. For functional imaging experiments and experiments involving acute slices prepared from mice expressing GCaMP5, mice were injected with AAV2/1-*ef1*α-GCaMP5 before the glass cover slip

was positioned. In both structural and functional imaging experiments, mice were allowed to recover for at least 18 days before the commencement of the imaging period. Monocular enucleation involved topical application of lidocaine to the area around the left eye under anaesthesia induced by ketamine-xylazine injection (dose as above) prior to surgical removal of the eye. Control 'sham-enucleated' mice were given time-matched anaesthesia. For functional imaging, we used intrinsic signal imaging to localize the monocular visual cortex as described previously (Keck et al., 2013). For functional imaging, brief isoflurane sedation (approximately 10 seconds) was used to head-fix the mouse. The animal was then allowed to habituate to the setup before data acquisition commenced.

***In vivo* structural and functional imaging**

Structural data were collected using repeated two-photon imaging (Denk et al., 1990). The illumination source was a Spectra Physics MaiTai BB laser with a DeepSee prechirp unit (Newport/Spectra Physics) set to 909 nm. Excitation and emitted light were delivered and collected through an Olympus 40× 0.8 NA water immersion objective. The average laser power delivered to the brain was < 50 mW. Scanning and image acquisition were conducted using Scanimage freeware (VidrioTech). Image acquisition parameters were: 64 × 64 μm field of view, 512 × 512 pixels, 0.5 μm step in depth between frames.

Functional calcium imaging was performed with a custom built two-photon microscope. The illumination source was a MaiTai eHP laser with a DeepSee prechirp unit (Newport/Spectra Physics) or a Chameleon Vision S (Coherent). The excitation wavelength was set to 910 nm. The scanhead was based on an 8 kHz resonant scanner

(Cambridge Technology), used in bidirectional mode. This enabled frame rates of 40 Hz at 400 × 600 pixels. A high power objective Z-piezo stage (Physik Instrumente) was used in order to move the objective down in steps of approximately 20 μm between frames and return to the initial position after four frames. With this system, we acquired data at four different depths, reducing the effective frame rate from 40 Hz to 10 Hz. Laser power was < 50 mW. We used a Nikon 16× 0.8 NA objective. Data were acquired with a 250 MHz digitizer (National Instruments) and pre-processed with a custom programmed FPGA (National Instruments).

Treadmill and visual stimulation

The awake paradigm, treadmill and visual stimuli have been described previously (Dombeck et al., 2007; Keller et al., 2012). Briefly, head-fixed animals ran on a spherical treadmill while visual stimuli were presented on two screens arranged at 60 degrees relative to each other in front of the mouse. This arrangement is intended to simulate visual flow similar to that experienced when running between two walls. Visual stimuli were full field gratings, the motion of which was controlled by rotation of the spherical treadmill. Experiments consisted of alternating 3 minute blocks in which the mouse either received coupled visual feedback or the screens were switched off. Each condition was repeated twice, followed by drifting gratings in eight directions (0-360 degrees by 45 degree steps) presented in a random order, with a spatial frequency of 0.04 cycles/degree and a temporal frequency of 2 Hz. The same visual stimulus paradigm was used at each time point before and after enucleation.

Analysis of structural imaging data

In vivo two-photon images of dendritic structures were analyzed in ImageJ (US National Institutes of Health) and custom written software in MATLAB (Mathworks). All analysis was done blind to the experimental condition on raw data in three-dimensions. To calculate spine density, all spines that were clearly distinguishable in any imaging plane (including z) were counted. Density measurements were made from 31 branches (20 deprived and 11 control). Spine size was calculated as previously described (Hofer et al., 2009; Keck et al., 2013). Briefly, only spines that extended from the dendrite in the x-y plane were included. The spine intensity was background subtracted and normalized by the intensity of the adjacent dendrite to account for differences in overall intensity between imaging sessions. Only spines present at all time points were included in the analysis of size. The survival fraction was calculated as the fraction of spines that were present at the time point -1 hour prior to enucleation that were still present at each subsequent time point after enucleation.

Analysis of functional imaging data

Analysis of functional imaging data was conducted as described previously (Keck et al., 2013; Keller et al., 2012). Briefly, data were full-frame registered using a custom written registration algorithm. Cells were selected based on mean and maximum projections of the data by hand (typically the nucleus was excluded from the selection) and using high-resolution depth stacks to ensure that cells did not have a filled nucleus (Fig. S2G,H). Use of the maximum projection ensured the inclusion of all the active cells, even those that were not visible in the mean projection. Note however, that this biased our cell selection towards active cells. Fluorescence traces were calculated as the average fluorescence of pixels lying within the cell in each

frame. To remove slow signal changes in raw fluorescence traces, the 8-percentile value of the fluorescence distribution in a ± 15 second window was subtracted from the raw fluorescence signal. $\Delta F/F_0$ signals were calculated by dividing the raw fluorescence signal by the median of each cell's fluorescence distribution. Cellular activity was calculated using integrated fluorescence as described previously (Keck et al., 2013).

Classification of visually responsive neurons

Neurons were considered visually responsive if they had significantly more activity during visual stimulation (light) than in darkness, in the absence of locomotion. These neurons accounted for 20% of the acquired neurons (215/1081), but their activity constituted 87% of the total activity of all the acquired neurons, under all recording conditions. GCaMP5 detection limits are several action potentials per second with saturation at a few tens of action potentials per second (Akerboom et al., 2012). Visually driven responses below these rates may not be detected in our measurements. Activity was normalized to the average of the two baseline sessions for individual cells. The day-to-day variability of neurons in control animals was calculated as the percentage of neurons that was active (or inactive) on a given day, but not active (or inactive) on the subsequent imaging day. Following sensory deprivation, cells that were not active in the last 72 hour imaging session were classified as inactive, other cells were considered recovering. Orientation selectivity was calculated as previously described (Ko et al., 2011), and all orientations and directions were presented prior to and following enucleation to account for any potential changes in selectivity. In figure S2D, visually evoked responses occurred within 500ms of the onset of the stimuli.

The frequency of the random responses was measured by superimposing the timing of the onset of the visual stimuli on periods when the animal was in darkness.

Classification of excitatory and inhibitory neurons

We sectioned and reconstructed the neurons previously imaged *in vivo* and performed immunohistochemistry to identify their cell type as GABA positive or GABA negative (Kerlin et al., 2010). At the end of the functional imaging experiment, we injected fluorescent beads adjacent to the imaged cortical regions. Animals were then sacrificed and the brain was fixed in 4% paraformaldehyde (PFA). In a separate set of mice, we injected the same AAV virus used in our functional imaging experiments, sacrificed the animals and performed immunohistochemistry to determine which specific inhibitory subtypes (i.e. somatostatin (SST), vasoactive intestinal peptide (VIP), calbindin (CB), reelin, parvalbumin (PV), calretinin (CR)) were labeled by our AAV virus. We found that all subtypes tested were labeled by our AAV virus (of GCaMP5 positive inhibitory cells SST 4%, VIP 12 %, CB 16%, reelin 16%, PV 14%, CR 12%). The following antibodies were used at the given concentrations: guinea-pig anti-GABA (1:500, Abcam), chicken anti-GFP (1:500, Millipore), mouse anti-parvalbumin (1:500, Swant), rabbit anti-calbindin (1:1000, Swant), goat anti-calretinin (1:500, Swant), mouse anti-reelin (1:250, MBLI), rabbit anti-VIP (1:200, Immunostar), rat anti-somatostatin (1:500, Millipore).

We used an adapted version of a previously described protocol for immunohistochemistry (Kreczko et al., 2009). Horizontal brain slices (80-100 μm) were prepared from the visual cortex, washed 4 times in PBS (pH 7.4) and then incubated overnight at -20 ºC in 30% sucrose (in PBS pH 7.4). The following day

sections were defrosted at room temperature and then rinsed 4 times (5 minutes each) in PBS. Sections were next transferred to -20 ºC methanol for 10 minutes before being rinsed 4 times (5 minutes each) in PBS. Sections were incubated in blocking serum (10% normal goat serum or 10% donkey serum, as appropriate) for two hours at room temperature. Blocking serum was then replaced with primary antibody (concentrations as above, in blocking serum) and slices incubated for one week at 4˚C. Following primary incubation, slices were washed three times (for 20 minutes each) with PBS containing 0.25% triton-X. Secondary antibodies were then applied to slices (diluted 1:500 in appropriate blocking serum: goat anti-mouse Alexa Fluor (AF) 633 (Invitrogen), goat anti-rabbit AF 405 (Invitrogen), goat anti-rat AF 594 (Abcam), donkey anti-goat AF 555 (Abcam), goat anti-guinea pig AF 633 (Invitrogen), goat anti-chicken AF 488 (Invitrogen)) for two hours at room temperature. Next, sections were washed three times in PBS (15 minutes) before mounting in Mowiol (Calbiochem). Slices were imaged using a confocal microscope (Nikon) with a Nikon 60× 1.4 NA oil immersion objective.

Imaged regions were tiled using ImageJ to reconstruct the functionally imaged cortical regions, using the fluorescent beads for guidance. Neurons were mapped to the *in vivo* images from the functional imaging experiments by three independent experimenters or by using a custom written algorithm in Matlab. The presence of the GABA antibody in neurons in an identified region was quantified as a ratio of somatic signal intensity over background intensity, so that GABA positive neurons had a 20% greater somatic labeling than background levels. Cells were determined to be GABA positive or negative without knowledge of the *in vivo* activity profiles.

The calcium transient kinetics of known inhibitory and excitatory neurons were then used to train a regression tree classification algorithm. To prevent any contamination from colliding calcium transients, we used isolated calcium events to train the classifier, which consisted of a mean of 13 ± 1 events per cell. We distinguished these isolated events by taking the first derivative of the smoothed calcium transient and identified a positive peak (with a threshold of 10% above baseline) that was not followed by a second positive peak for 10 seconds. Calcium transient shape parameters were first normalized to the mean of all cells in a given animal, then these values were log-normalized. We trained the classification tree on a randomly selected half of the dataset and then cross-validated it on the other half of the dataset, which was novel to the classifier. Performance on this novel dataset was 91% correct.

c-Fos immunohistochemistry

We sectioned and reconstructed the neurons previously imaged *in vivo* to use immunohistochemistry to identify GCAMP5 positive neurons as either c-Fos positive or c-Fos negative. At the end of the functional imaging experiment 72 hours after enucleation, we injected fluorescent beads adjacent to the imaged cortical regions. Acute horizontal slices of visual cortex were prepared from these animals and after recovery for four hours in artificial cerebral spinal fluid (ACSF) with 1 μM tetrodotoxin (TTX) saturated with 95% O_2 / 5% CO_2, the slices were moved to 1% PFA for 24 hours and then incubated overnight at 4 ºC in mouse anti-c-Fos (1:500, Santa Cruz). Following primary incubation, slices were washed three times (for 20 minutes each) with PBS containing 0.25% triton-X. Slices were then incubated for two hours at room temperature in the anti-mouse AF 568 secondary antibody (1:1000, Invitrogen). For slices following electrophysiology experiments the secondary

antibody was changed to anti-mouse AF 488 (1:1000, Invitrogen). After this, sections were washed three times in PBS (15 minutes) before mounting in Mowiol (Calbiochem). Slices were imaged and reconstructed as described above for GABAergic reconstructions.

c-Fos Quantification

The presence of c-Fos antibody in neurons in an identified region was quantified as a ratio of somatic signal intensity over background intensity, so that c-Fos positive neurons had a somatic signal that was at least 20% greater than background levels. Cells were classified as c-Fos positive or c-Fos negative blind to the *in vivo* activity profile for that cell. In a subset of experiments to quantify the density of c-Fos positive neurons, we counted the number of c-Fos positive cells in a known volume of visual cortex.

Measuring neuronal correlations

We computed the pairwise correlations between calcium traces of all active neurons in the same cortical region. Cells were considered to be correlated if the correlation coefficient was positive and statistically significant ($p<0.05$). The average and summed correlation strength, as well as the percentage of correlated neurons was calculated from statistically significant correlations. Persistent correlations were those that were significant both in the baseline and 72 hours after (sham) enucleation.

Electrophysiology

Deeply anaesthetized mice were transcardially perfused with 10 ml of 4 ºC dissection ACSF (in mM, 108 choline-Cl, 3 KCl, 26 $NaHCO_3$, 1.25 $NaHPO_4$, 25 D-glucose, 3

Na pyruvate, 2 $CaCl_2$ and 1 $MgSO_4$ saturated with 95% O_2 / 5% CO_2). Coronal brain slices 300 μm thick were prepared (Vibratome 3000, Leica) from visual cortex. Slices were incubated for at least 60 minutes in a holding chamber before recordings were made at room temperature (24 ºC) in a recording ACSF (in mM, 126 NaCl, 3.5 KCl, 25 $NaHCO_3$, 1 $NaHPO_4$, 25 D-glucose, 2 $CaCl_2$ and 1 $MgSO_4$ saturated with 95% O_2 / 5% CO_2). Recordings were targeted to monocular visual cortex based on stereotaxic coordinates. We recorded from L2/3 cells on a custom built microscope under infrared differential interference contrast microscopy. L2/3 pyramidal neurons were identified based on spiking properties and pyramidal shaped soma and recorded in voltage or current clamp mode, as appropriate (Multiclamp 700B, Molecular Devices), using Ephus freeware acquisition software (VidrioTech). In a subset of experiments, we reconstructed inhibitory cells and immunolabeled GABA to confirm the cell type. We did not correct for the liquid junction potential. Patch pipettes (4 – 7 MΩ) contained the following in mM: 130 $KMeSO_4$, 8 NaCl, 2 KH_2PO_4, 2 D-glucose and 10 HEPES. mEPSP recordings were made at resting membrane potential with 1 μM TTX and 10 mM Gabazine in the bath solution. Recordings were discarded if the series resistance varied by ± 15% or if the resting membrane potential or input resistance varied by ± 10% across the recording period. mEPSP recordings were filtered at 3 kHz and digitized at 20 kHz and analyzed blind to the experimental condition in the MiniAnalysis programme (Synaptosoft) according to previous criteria (Clements and Bekkers, 1997) so that the smallest detected event was 2.5 times the root mean square of the baseline noise. mIPSCs and mEPSCs were collected and analyzed as described previously (Keck et al., 2013). The ratio of the inhibitory to excitatory drive at L2/3 excitatory neurons was assessed in whole-cell mode using stimulation of horizontal L2/3 inputs with a concentric bipolar stimulating electrode.

L2/3 neurons were held at either – 70 mV or +10 mV and extracellular stimulation consisted of 1 ms current pulses with a stimulation intensity set to 50% of the intensity that evoked the maximum EPSP amplitude. Neuronal excitability was recorded in the presence of bath-applied synaptic blockers (20 μM 6-cyano-7-nitroquinoxaline2,3-dione (CNQX), 50 μM 2-amino-5-phosphonopentanoate acid (APV), and 10 mM Gabazine) by somatic injection of a 500 ms depolarizing current step in increments of 10 pA. The slope was calculated from the linear component of the input-output function. Passive membrane properties were estimated from -100 pA current injections, based on the membrane deflection and time-course of decay to baseline membrane potential. For c-Fos excitability experiments, current injection was increased in 10 pA increments until a single action potential was recorded, at which point the pipette was withdrawn. For all c-Fos electrophysiology experiments, following recording, the slice was fixed overnight in 1% PFA and immunohistochemistry was performed the following day.

Statistics

All statistical analysis was performed either in Matlab or SigmaPlot v.12. Comparisons were made using parametric (t-test, ANOVA with Holm-Sidak post-hoc test, repeated measures ANOVA with post-hoc test) or non-parametric (Mann-Whitney Rank Sum, ANOVA on ranks) statistics where appropriate. Correlation coefficients were calculated with a Spearman's rank correlation coefficient.

References

Akerboom, J., Chen, T.-W., Wardill, T.J., Tian, L., Marvin, J.S., Mutlu, S., Calderón, N.C., Esposti, F., Borghuis, B.G., Sun, X.R., et al. (2012). Optimization of a GCaMP calcium indicator for neural activity imaging. J. Neurosci. *32*, 13819–13840.

Clements, J.D., and Bekkers, J.M. (1997). Detection of spontaneous synaptic events with an optimally scaled template. Biophys. J. *73*, 220–229.

Denk, W., Strickler, J.H., and Webb, W.W. (1990). Two-photon laser scanning fluorescence microscopy. Science *248*, 73–76.

Dombeck, D.A., Khabbaz, A.N., Collman, F., Adelman, T.L., and Tank, D.W. (2007). Imaging large-scale neural activity with cellular resolution in awake, mobile mice. Neuron *56*, 43–57.

Feng, G., Mellor, R.H., Bernstein, M., Keller-Peck, C., Nguyen, Q.T., Wallace, M., Nerbonne, J.M., Lichtman, J.W., and Sanes, J.R. (2000). Imaging neuronal subsets in transgenic mice expressing multiple spectral variants of GFP. Neuron *28*, 41–51.

Hofer, S.B., Mrsic-Flogel, T.D., Bonhoeffer, T., and Hübener, M. (2009). Experience leaves a lasting structural trace in cortical circuits. Nature *457*, 313–317.

Holtmaat, A., Bonhoeffer, T., Chow, D.K., Chuckowree, J., De Paola, V., Hofer, S.B., Hübener, M., Keck, T., Knott, G., Lee, W.-C.A., et al. (2009). Long-term, high-resolution imaging in the mouse neocortex through a chronic cranial window. Nat. Protoc. *4*, 1128–1144.

Keck, T., Keller, G.B., Jacobsen, R.I., Eysel, U.T., Bonhoeffer, T., and Hübener, M. (2013). Synaptic scaling and homeostatic plasticity in the mouse visual cortex in vivo. Neuron *80*, 327–334.

Keller, G.B., Bonhoeffer, T., and Hübener, M. (2012). Sensorimotor mismatch signals in primary visual cortex of the behaving mouse. Neuron *74*, 809–815.

Kerlin, A.M., Andermann, M.L., Berezovskii, V.K., and Reid, R.C. (2010). Broadly tuned response properties of diverse inhibitory neuron subtypes in mouse visual cortex. Neuron *67*, 858–871.

Ko, H., Hofer, S.B., Pichler, B., Buchanan, K.A., Sjöström, P.J., and Mrsic-Flogel, T.D. (2011). Functional specificity of local synaptic connections in neocortical networks. Nature *473*, 87–91.

Kreczko, A., Goel, A., Song, L., and Lee, H.-K. (2009). Visual deprivation decreases somatic GAD65 puncta number on layer 2/3 pyramidal neurons in mouse visual cortex. Neural Plast. *2009*, 415135.
